# Expression of epigenetic machinery genes is sensitive to maternal obesity and weight loss in relation to fetal growth in mice

**DOI:** 10.1186/s13148-016-0188-3

**Published:** 2016-02-27

**Authors:** Polina E. Panchenko, Sarah Voisin, Mélanie Jouin, Luc Jouneau, Audrey Prézelin, Simon Lecoutre, Christophe Breton, Hélène Jammes, Claudine Junien, Anne Gabory

**Affiliations:** UMR BDR, INRA, ENVA, Université Paris Saclay, 78350 Jouy en Josas, France; Ecole Doctorale 394 “Physiologie, physiopathologie et thérapeutique”, Université Pierre et Marie Curie, 75252 Paris, France; Univ. Lille, EA4489, Équipe Malnutrition Maternelle et Programmation des Maladies Métaboliques, F59000 Lille, France; Université Versailles Saint-Quentin en Yvelines (UVSQ), Guyancourt, France

**Keywords:** Maternal obesity, Preconceptional weight loss, Fetal growth restriction, Epigenetic machinery, Histone deacetylases (HDACs), Lysine acetyltransferases (KATs), Placenta, Liver

## Abstract

**Background:**

Maternal obesity impacts fetal growth and pregnancy outcomes. To counteract the deleterious effects of obesity on fertility and pregnancy issue, preconceptional weight loss is recommended to obese women. Whether this weight loss is beneficial/detrimental for offspring remains poorly explored. Epigenetic mechanisms could be affected by maternal weight changes, perturbing expression of key developmental genes in the placenta or fetus. Our aim was to investigate the effects of chronic maternal obesity on feto-placental growth along with the underlying epigenetic mechanisms. We also tested whether preconceptional weight loss could alleviate these effects.

**Results:**

Female mice were fed either a control diet (CTRL group), a high-fat diet (obese (OB) group), or a high-fat diet switched to a control diet 2 months before conception (weight loss (WL) group). At mating, OB females presented an obese phenotype while WL females normalized metabolic parameters. At embryonic day 18.5 (E18.5), fetuses from OB females presented fetal growth restriction (FGR; −13 %) and 28 % of the fetuses were small for gestational age (SGA). Fetuses from WL females normalized this phenotype. The expression of 60 epigenetic machinery genes and 32 metabolic genes was measured in the fetal liver, placental labyrinth, and junctional zone. We revealed 23 genes altered by maternal weight trajectories in at least one of three tissues. The fetal liver and placental labyrinth were more responsive to maternal obesity than junctional zone. One third (18/60) of the epigenetic machinery genes were differentially expressed between at least two maternal groups. Interestingly, genes involved in the histone acetylation pathway were particularly altered (13/18). In OB group, *lysine acetyltransferases* and *Bromodomain-containing protein 2* were upregulated, while most histone deacetylases were downregulated. In WL group, the expression of only a subset of these genes was normalized.

**Conclusions:**

This study highlights the high sensitivity of the epigenetic machinery gene expression, and particularly the histone acetylation pathway, to maternal obesity. These obesity-induced transcriptional changes could alter the placental and the hepatic epigenome, leading to FGR. Preconceptional weight loss appears beneficial to fetal growth, but some effects of previous obesity were retained in offspring phenotype.

**Electronic supplementary material:**

The online version of this article (doi:10.1186/s13148-016-0188-3) contains supplementary material, which is available to authorized users.

## Background

The worldwide prevalence of obesity in women was 38 % in 2013 [1]. Obesity during pregnancy comprises increased risks for metabolic and obstetrical complications (e.g., gestational hypertension and diabetes, and preeclampsia) but also stillbirth, prematurity, and congenital malformations [2]. Fetal growth could be particularly impacted by maternal obesity. Maternal obesity is associated with macrosomia or, on the contrary, with fetal growth restriction (FGR) [3–6]. FGR is associated with a high incidence of metabolic diseases in adulthood [7, 8], which is consistent with the *developmental origins of health and disease* (DOHaD) concept. This concept, also named “developmental programming” or “conditioning,” states that environmental factors during early development could predispose an individual to chronic diseases [9].

Despite the high incidence of FGR in obese women, little is known about the underlying mechanisms. FGR could result from insufficient oxygen supply due to disturbed vascularization, increased lipid accumulation, and macronutrients transport in the placenta, crucial organ regulating appropriate fetal development [10–12]. Importantly, different placental parts have different functions and cellular populations [13]. The labyrinth is a zone of active exchange between maternal and fetal blood, while the junctional zone provides hormone production and storage of nutrients that are necessary for fetal development [13]. The structure and the function of several organs could be affected in the offspring of obese mothers. In utero alterations of hepatic development and function by maternal obesity could disturb metabolic homeostasis [14, 15]. The effect of obesity on organogenesis and gene expression in growth-restricted fetuses needs further investigation; the current efforts are indeed focused on FGR induced by poor maternal nutrition [16].

Modulation of offspring phenotype in response to maternal environment could be mediated by epigenetic mechanisms. Epigenetic marks (e.g., DNA methylation, histone posttranslational modifications) are stable but reversible covalent modifications that are regulated by a complex epigenetic machinery. Its actors “write,” “erase” or “read” epigenetic marks, establishing the epigenome of the cell in conjunction with environmental factors. This epigenetic landscape is dynamic during development and controls gene expression patterns in a tissue-specific manner. Alterations of DNA methylation, histone modifications and, to a lesser extent, of their regulators were observed in relation to FGR caused by different factors [17–22]. Epigenetic disturbances in growth-restricted fetuses in the context of maternal diet-induced obesity need further investigation. Maternal unbalanced nutrition and metabolic state could impact certain epigenetic enzymes in the developing organs of the offspring, affecting the epigenome [23, 24]. As epigenetic marks can be transmitted through generations of cell divisions, epigenetics has emerged as a plausible mechanism for long-term memory of environmental insults [23, 25].

To counteract the negative effects of obesity on pregnancy outcomes, preconceptional weight loss is currently recommended to women with high body mass index [26]. Nevertheless, very few studies have assessed the consequences of maternal weight loss on fetal growth [27]. In humans, weight loss between two pregnancies reduces the risk of macrosomia [28]. However, in another cohort, weight loss between the age of 20 years and conception had a negative impact on birth weight [29]. Thus, the impact of maternal preconceptional weight changes on fetal growth and underlying epigenetic processes needs to be clarified. Currently, there is no relevant mouse model to study this important issue for public health.

Based on the observations that maternal obesity impairs feto-placental development, our aim was to examine the impact of maternal weight trajectories (obesity or weight loss) on the expression of epigenetic and metabolic genes in the fetal liver and in the placental labyrinth and junctional zone. We showed that maternal obesity induced FGR, which was associated with an altered expression of histone acetylation modifiers in the fetal liver and labyrinth, but not in the junctional zone. In contrast, correction of obesity during the preconceptional period by nutritional intervention normalized fetal weight and induced an adaptation at the transcriptional level. This study provides a novel mouse model for investigating the molecular mechanisms of obesity-induced FGR and highlights the sensitivity of the epigenetic machinery to maternal nutrition and metabolism.

## Results

### High-fat diet (HFD) induced severe obesity in female mice; switching to a control diet (CD) induced weight loss and normalization of metabolic parameters

**Fig. 1:**
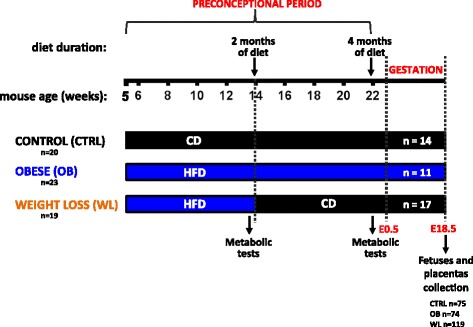
Experimental design. C57BL/6j females were fed a control diet (*CD*) or a high-fat diet (*HFD*) throughout the study. After 2 months of diet, HFD was switched to CD for the weight loss group. After 2 and 4 months of diet, females’ metabolic parameters (cholesterol, fasting glucose levels, and glucose tolerance) were measured. Females were mated with males on a standard diet and sacrificed at E18.5, then fetuses and placentas were weighed and collected

**Fig. 2:**
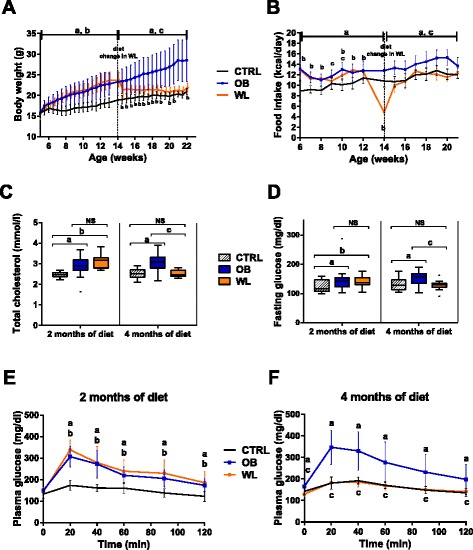
Body weight and metabolic parameters of OB and WL females during the preconceptional period. **a** Body weight. (*a*) *P* < 0.05 OB vs. CTRL, (*b*) *P* < 0.05 WL vs. CTRL, (*c*) *P* < 0.05 WL vs. OB. *n* = 20 CTRL, 23 OB, 19 WL. **b** Caloric intake. (*a*) *P* < 0.001 OB vs. CTRL, (*b*) *P* < 0.001 WL vs. CTRL, (*c*) *P* < 0.05 WL vs. OB. *n* = 18–20 CTRL, 23 OB, 17–19 WL. **c** Plasma cholesterol levels after 2 and 4 months of preconceptional diet. *NS* nonsignificant. (*a*) *P* < 0.001 OB vs. CTRL, (*b*) *P* < 0.001 WL vs. CTRL, (*c*) *P* < 0.001 WL vs. OB. *n* = 17–19 CTRL, 22–23 OB, 19 WL. **d** Fasting glucose level after 2 and 4 months of preconceptional diet. *NS* nonsignificant. (*a*) *P* < 0.05 OB vs. CTRL, (*b*) *P* < 0.05 WL vs. CTRL, (*c*) *P* < 0.05 WL vs. OB. *n* = 17–19 CTRL, 23 OB, 19 WL. **e** Plasma glucose levels during oral glucose tolerance test (OGTT) after 2 months of diet. (*a*) *P* < 0.001 OB vs. CTRL, (*b*) *P* < 0.001 WL vs. CTRL, 11–12 females per group. **f** Plasma glucose levels during OGTT after 4 months of diet. (*a*) *P* < 0.001 OB vs. CTRL, (*c*) *P* < 0.001 WL vs. OB, 12 females per group. Data are expressed as mean ± St. Dev (*a*, *b*, *e*, *f*) or as Tukey’s boxplot (*c*, *d*)

Female mice were fed either a CD (control females (CTRL)) or a HFD (obese females (OB)) for 4 months during the preconceptional period (Fig. 1). From the start of the diet and up to mating, OB females put on weight faster (*β* = 0.52, *P* < 0.001) and were heavier than CTRL females on a CD (*P* < 0.001, Fig. 2a). OB females weighed 27 and 36 % more than CTRL females after 2 and 4 months of diet, respectively. After 2 months on a HFD, we replaced HFD by CD for a subset of the OB females to induce weight loss (WL females) (Fig. 1). The weight of WL females was lower than the weight of OB females as early as 3 days after CD initiation and afterwards (*P* < 0.05 at all time points, Fig. 2a). WL females tended to normalize their weight (*P* = 0.105, week 19.5, *P* = 0.062, week 21, *P* = 0.051, week 21.5, *P* = 0.031, week 22, WL vs. CTRL). However, they remained 5 % heavier than CTRL females before mating.

The caloric intake of OB females was higher than the caloric intake of CTRL females during 4 months of preconceptional diet (*P* < 0.001, Fig. 2b) but OB and CTRL females had a similar food intake to body weight ratio (kcal/kg/day) (Additional file 1: Figure S1). WL females drastically decreased their caloric intake right after the nutritional intervention (*P* < 0.001, WL vs. CTRL/OB; Fig. 2b) and normalized this parameter 1 week later (*P* = 0.15, week 15, WL vs. CTRL; *P* < 0.05, WL vs. OB) and thereafter. Metabolic parameters were assessed after 2 and 4 months of diet (Fig. 1). OB females were hypercholesterolemic, hyperglycemic, and glucose intolerant compared to CTRL females (*P* < 0.001, *P* < 0.05, and *P* < 0.001 at each time point, respectively, Fig. 2c–f). The nutritional intervention normalized these parameters in WL females.

In summary, OB females were obese and had an impaired glucose metabolism and hypercholesterolemia. Switching to a CD allowed complete restoration of all these parameters although WL females remained 5 % heavier than CTRL females before mating.

### Obese dams on a HFD gained less weight at term of pregnancy

**Fig. 3:**
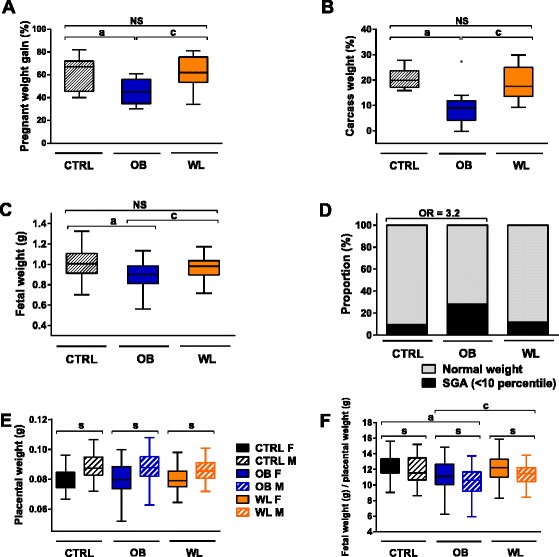
Body weight and fetal and placental weights in OB and WL dams at E18.5. **a** Dams pregnant body weight gain (percentage of initial weight). *NS* nonsignificant. (*a*) *P* < 0.001 OB vs. CTRL. (*c*) *P* < 0.001 WL vs. OB. *n* = 13 CTRL, 11 OB, 17 WL. **b** Dams carcass weight (percentage of initial weight) at sacrifice. *NS* nonsignificant. (*a*) *P* < 0.001 OB vs. CTRL, (*c*) *P* < 0.001 WL vs. OB. *n* = 13 CTRL, 10 OB, 16 WL. **c** Fetal weight. *NS* nonsignificant. (*a*) *P* < 0.001 OB vs. CTRL. (*c*) *P* < 0.001 WL vs. OB. Data from males and females were combined as there was no effect of sex on fetal weight. *n* = 75 CTRL, 74 OB, 119 WL. **d** Proportion of small for gestational age (SGA) fetuses. Data from males and females were combined as there was no effect of sex on fetal weight. CTRL (*n* = 7 SGA/75 fetuses), OB (*n* = 21/74), WL (*n* = 14/119). Maternal obesity: odds ratio (OR) of being SGA = 3.2 (95 % CI 1.19–9.76, *P* = 0.028). **e** Placental weight. (*s*) *P* < 0.001 males vs. females. *n* = 36 CTRL F, 39 CTRL M, 35 OB F, 39 OB M, 61 WL F, 58 WL M. **f** Fetal-weight-to-placental weight ratio index. (*a*) *P* = 0.001 OB vs. CTRL. (*c*) *P* < 0.001 WL vs. OB. (*s*) *P* = 0.001 males vs. females. *n* = 36 CTRL F, 39 CTRL M, 35 OB F, 39 OB M, 61 WL F, 58 WL M

To determine the potential effects of maternal obesity and preconceptional weight loss on fetal outcomes, we mated CTRL, OB, and WL females after 4 months of preconceptional diet with males on a standard laboratory diet (Fig. 1). Preconceptional diet was maintained during pregnancy. OB dams had reduced total pregnant body weight gain and carcass weight compared with CTRL and WL dams at embryonic day 18.5 (E18.5) (*P* < 0.001, Fig. 3a, b). There were no differences between WL and CTRL dams (*P* = 0.83 for total weight gain; *P* = 0.56 for carcass weight). The maternal group explained 29 % of the variance in total body weight gain and 37 % of the variance in carcass weight at E18.5. OB and WL dams had increased litter size vs. CTRL dams at E18.5 (*P* < 0.001; CTRL 5.5 ± 2.18, *n* = 14 litters; OB 6.8 ± 1.99, *n* = 11 litters; WL 7.0 ± 2.26, *n* = 17 litters). There was no difference in litter size between OB and WL dams (*P* = 0.13).

### Maternal obesity induced fetal growth restriction, while preconceptional weight loss allowed restoration of fetal weight

As several studies identified sex-specific effects of maternal obesity on fetuses and placentas, we tested the effect of sex on fetal and placental weights [12, 30–32]. Sex did not affect fetal weight (*P* = 0.17) but affected placental weight and fetal-weight-to-placental-weight ratio index (FPI). Male placentas were heavier than female placentas (*P* < 0.001; difference in CTRL 11 %, OB 10 %, and WL 7 %), and FPI was lower in males than in females for all maternal groups (*P* < 0.001). We therefore adjusted for sex in the placental weight and FPI analysis only. Moreover, litter size affected fetal and placental weights (*P* < 0.001), and even if it explained only 4.6 and 7.7 % of the variance, respectively (Additional file 1: Figure S2 and S3), we adjusted all analyses for this parameter. There were no differences in the mother’s age at mating between the three investigated groups (CTRL 26.1 ± 1.94 weeks; OB 25.6 ± 1.83 weeks; WL 25.6 ± 1.71 weeks). Maternal age/diet duration correlated with fetal weight and FPI but not with placental weight (Pearson’s correlation test, *r* = 0.39, *P* < 0.001, *r* = 0.40, *P* < 0.001, *r* = −0.08, *P* = 0.17, respectively, Additional file 1: Figure S4). All studies were therefore adjusted for maternal age, which was also equivalent to diet duration in our experimental protocol.

We then examined the effect of maternal metabolism on fetal and placental weights at E18.5. We observed a 13 % reduction of the weight in fetuses of OB dams compared with CTRL dams (*P* < 0.001, Fig. 3c). Fetuses of WL dams had similar weight to fetuses of CTRL dams (*P* = 0.17, Fig. 3c) but heavier than fetuses of OB dams (*P* < 0.001). Overall, maternal group and age explained 15 and 14 % of the variance in fetal weight, respectively. We determined the proportion of small for gestational age (SGA) fetuses, defined as fetal weight <10th percentile of CTRL population. There were 28.4 % of SGA fetuses in OB dams and 11.8 % in the WL dams (Fig. 3d). The odds of being SGA was increased in OB group by a factor of 3.2 (logistic regression, 95 % CI 1.19–9.76, *P* = 0.028). In WL group, the odds of being SGA was not altered compared to CTRL group (*P* = 0.48). Increase in maternal age/diet duration for 1 week decreased the odds by a factor of 0.61 (*P* < 0.001). Gaussian distributions of fetal weight in the three maternal groups are available in Additional file 1: Figure S5.

There was no effect of maternal group on placental weight at E18.5 (*P* = 0.42, Fig. 3e). FPI, which represents placental efficiency, was reduced in the fetuses of OB dams compared with those of CTRL and WL dams (*P* < 0.001, Fig. 3f). However, WL and CTRL dams had similar FPI (*P* = 0.16). Maternal group explained 12 % of the variance in FPI. In female fetuses, there was a correlation between fetal and placental weight in all maternal groups (Pearson’s correlation test, adjusting for factor “mother”: CTRL *r* = 0.50, *P*_adj_ = 0.006; OB *r* = 0.49; *P*_adj_ = 0.006; WL *r* = 0.36, *P*_adj_ = 0.0063; Additional file 1: Figure S5). In male fetuses, we observed a correlation in WL (*r* = 0.45, *P*_adj_ = 0.002), but not in CTRL and OB groups (*r* = 0.28, *P*_adj_ = 0.097 and *r* = 0.1, *P*_adj_ = 0.54, respectively).

Thus, maternal chronic obesity caused FGR in both sexes and impaired placental efficiency. Preconceptional weight loss induced by nutritional intervention abolished this FGR and restored placental efficiency.

### Maternal obesity altered gene expression in the fetal liver and placental labyrinth, but not in junctional zone

To unravel the molecular mechanisms of the impact of maternal obesity and preconceptional weight loss in growth-restricted offspring, we assessed the gene expression at E18.5 using custom TaqMan low-density arrays (TLDAs). We tested the expression of 60 epigenetic machinery genes and 32 genes involved in metabolism or in development (Additional file 2: Table S1). Based on the literature and our previous studies, these epigenetic genes were selected because of their implication in metabolic processes and obesity or type 2 diabetes [30, 33]. Some of the metabolic genes assessed in our study are known targets of developmental conditioning, and for a subset of these genes, the epigenetic alterations are documented in this context. A description of the selection criteria of genes for the custom TLDA design is available in Additional file 3.

The vast majority of expression studies are performed in whole placentas, but epigenetic and metabolic processes may not be the same in different placental layers since they have different functions and cell populations [30, 34, 35]. The liver is a major organ regulating the metabolic processes and it is particularly affected by obesity [14, 15]. Thus, the expression study was performed in placental labyrinth and junctional zone separately, as well as in the fetal liver, as we also aimed to evaluate the impact of maternal nutrition on fetal tissues.

**Fig. 4:**
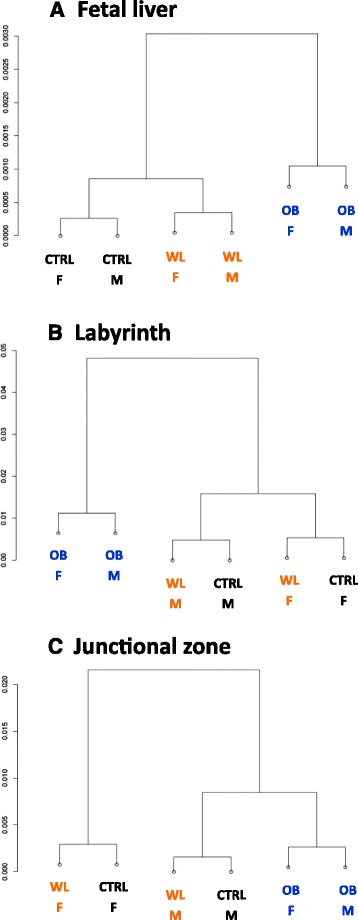
Hierarchical clustering of gene expression in the fetal liver, placental labyrinth, and junctional zone. The clustering is based on the expression of 80 genes in the liver (**a**), 86 genes in the labyrinth (**b**), and 89 genes in the junctional zone (**c**) in CTRL, OB, and WL females. Data are represented as Pearson’s correlation distance. Non-amplified genes were removed from analysis. *F* females, *M* males

Hierarchical clustering based on mean gene expression revealed that gene expression was affected by maternal diet in the liver and labyrinth: the OB group clustered away from the CTRL and WL groups (Fig. 4a, b). The effect of obesity was weaker in the junctional zone as OB males and females clustered with WL and CTRL males (Fig. 4c). In the liver, groups clustered according to maternal diet while in placental layers, CTRL and WL groups clustered according to fetal sex. Thus, maternal obesity affected the mean expression of all tested genes in the fetal liver and labyrinth, while maternal weight loss restored it.

We showed that 23 genes were significantly altered by maternal weight trajectories in at least one of three tissues (Table 1). The full list of mean expression level per gene and per group and adjusted *p* values are presented in Additional file 4: Table S2.

**Table 1:** Differentially expressed genes in the fetal liver, placental labyrinth, and junctional zone at E18.5

Tissue	Gene	CTRL group	OB group	WL group	*P* value		
					OB vs. CTRL	WL vs. CTRL	WL vs. OB
Fetal liver	*Kat2a* (*Gcn5*)	2.92 ± 0.90	4.77 ± 1.48	4.14 ± 1.51	↗ 0.004	0.118	0.622
	*Kat3a* (*Crebbp*)	0.71 ± 0.19	1.14 ± 0.34	1.04 ± 0.37	↗ 0.008	0.055	0.745
	*Kat6b* (*Myst4*)	0.97 ± 0.29	1.65 ± 0.48	1.26 ± 0.41	↗ 0.004	0.209	0.209
	*Kat13d* (*Clock*)	2.19 ± 0.59	3.67 ± 0.89	3.16 ± 1.23	↗ <0.001	0.307	0.445
	*Hdac2*	0.43 ± 0.17	0.20 ± 0.07	0.26 ± 0.13	↘ 0.004	0.065	0.496
	*Hdac6*	1.44 ± 0.43	2.29 ± 0.71	1.78 ± 0.74	↗ 0.012	0.445	0.339
	*Brd2*	0.25 ± 0.07	0.34 ± 0.07	0.37 ± 0.10	↗ 0.039	0.024	0.731
	*Prmt1*	0.50 ± 0.15	0.78 ± 0.21	0.61 ± 0.23	↗ 0.008	0.431	0.279
	*Prmt7*	7.1 ± 2.6	11.6 ± 3.9	8.5 ± 3.0	↗ 0.016	0.496	0.166
	*Gck*	0.81 ± 0.56	0.26 ± 0.24	0.76 ± 0.61	↘ 0.031	0.940	0.088
	*Lepr*	0.22 ± 0.09	0.25 ± 0.07	0.17 ± 0.05	0.637	0.377	↘ 0.039
Labyrinth	*Kat1* (*Hat1*)	0.024 ± 0.011	0.041 ± 0.013	0.037 ± 0.010	↗ 0.012	↗ 0.029	0.463
	*Kat3a* (*Crebbp*)	1.22 ± 0.42	1.49 ± 0.43	1.84 ± 0.60	0.215	↗ 0.029	0.192
	*Kat3b* (*Ep300*)	0.012 ± 0.006	0.030 ± 0.014	0.029 ± 0.014	↗ 0.003	↗ 0.006	0.907
	*Kat13b* (*Ncoa3*)	0.045 ± 0.022	0.084 ± 0.033	0.087 ± 0.028	↗ 0.017	↗ 0.003	0.871
	*Hdac2*	0.35 ± 0.15	0.23 ± 0.15	0.18 ± 0.08	0.124	↘ 0.014	0.448
	*Hdac3*	4.03 ± 1.41	2.60 ± 0.62	3.81 ± 1.43	↘ 0.021	0.797	0.053
	*Hdac10*	0.22 ± 0.08	0.10 ± 0.05	0.22 ± 0.10	↘ 0.003	0.978	↗ 0.003
	*Sirt4*	0.32 ± 0.14	0.23 ± 0.07	0.37 ± 0.16	0.124	0.478	↗ 0.046
	*Brd2*	0.73 ± 0.26	0.91 ± 0.18	1.06 ± 0.31	0.124	↗ 0.036	0.266
	*Kmt1d* (*Ehmt1*)	2.09 ± 0.74	1.34 ± 0.37	2.50 ± 1.15	↘ 0.029	0.402	↗ 0.017
	*Mbd5*	0.031 ± 0.010	0.022 ± 0.007	0.035 ± 0.013	0.053	0.480	↗ 0.021
	*Mecp2*	0.035 ± 0.012	0.029 ± 0.010	0.045 ± 0.012	0.247	0.112	↗ 0.007
	*Hsd11β1*	49.7 ± 25.5	26.1 ± 13.8	52.1 ± 32.0	↘ 0.046	0.892	0.065
	*Irs1*	0.71 ± 0.36	0.38 ± 0.19	0.65 ± 0.41	↘ 0.043	0.777	0.124
	*Tph1*	0.021 ± 0.010	0.012 ± 0.006	0.021 ± 0.009	↘ 0.033	0.978	↗ 0.04951
Junctional zone	*Kat3b* (*Ep300*)	0.029 ± 0.008	0.042 ± 0.012	0.045 ± 0.012	0.101	↗ 0.040	0.743

Data are represented as mean expression levels ± St.Dev. When the *p* value was significant, an arrow showing the sense of variation was added (**↘** downregulation, **↗** upregulation)

The expression of *Kdm5d* (*Jarid1d*) and *Uty* genes, which are localized on the Y chromosome, was restricted to male samples. Their paralogs located on the X chromosome (*Kdm5c/Jarid1c* and *Kdm6a/Utx*), and which partially escape X inactivation, were not differentially expressed between males and females, except for *Kdm6a* in the junctional zone (*p* = 0.003). Two other genes showed sex differences in this tissue independent of maternal dietary group: *Bdnf* and *Lpl* were also more expressed in female (*P* = 0.039 and 0.033, respectively). We then pooled male and female data from the same maternal group to assess the effect of maternal diet on gene expression. Maternal age did not correlate with gene expression in any of the three investigated tissues (Pearson’s correlation test; *P* > 0.05 for all genes).

### Maternal obesity and weight loss altered the expression of epigenetic machinery genes in the fetal liver

**Fig. 5:**
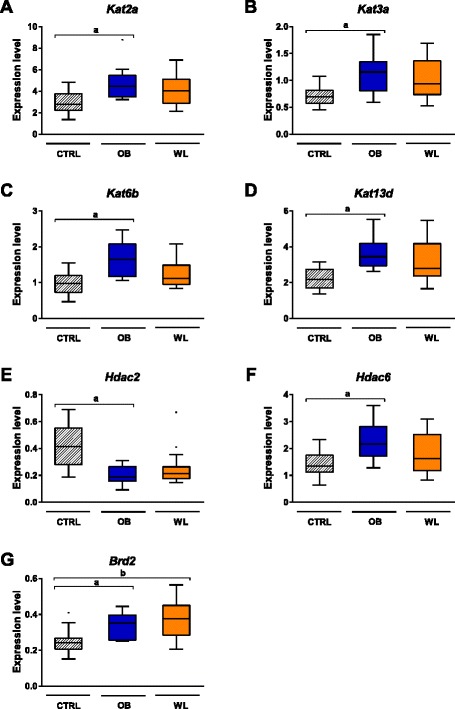
Genes implicated in histone acetylation are differentially expressed in the fetal liver at E18.5. We assessed the expression level of 60 genes of the epigenetic machinery using TaqMan low-density arrays. The expression of *Kat2a* (**a**), *Kat3a* (**b**), *Kat6b* (**c**), *Kat13d* (**d**), *Hdac2* (**e**), *Hdac6* (**f**), and *Brd2* (**g**) was affected by maternal obesity. (*a*) *P*_adj_ < 0.05 OB vs. CTRL, (*b*) *P*_adj_ < 0.05 WL vs. CTRL. CTRL (*n* = 16), OB (*n* = 14), WL (*n* = 16)

Maternal weight trajectories affected the transcription of nine epigenetic genes in the liver (Fig. 5, Table 1). Expression of the histone deacetylase *Hdac2* was reduced in OB fetuses compared to CTRL fetuses (Table 1, Fig. 5e). On the contrary, expression of the lysine acetyltransferases *Kat2a* (*Gcn5*), *Kat3a* (*Creb binding protein*), *Kat6b* (*Myst4*) and *Kat13d* (*Clock*), the arginine methyltransferases *Prmt1* and *Prmt7*, the histone deacetylase *Hdac6*, and the bromodomain protein *Brd2* was increased in fetuses from OB dams. Maternal weight loss induced various transcriptional responses of epigenetic machinery genes. On the one hand, *Brd2* expression remained increased in WL compared to CTRL fetuses, showing no normalization of its expression (Fig. 5g). Expression of *Hdac2* and *Kat3a* tended to remain altered in the liver of WL fetuses (*P*_adj_ = 0.065 and *P*_adj_ = 0.055, respectively, WL vs. CTRL). On the other hand, expression of *Kat2a*, *Kat6b*, *Kat13d*, *Hdac6*, *Prmt1*, and *Prmt7* was similar in WL and CTRL fetuses. Therefore, maternal weight loss restored the expression of some of the genes that were altered by maternal obesity, but not of all.

**Fig. 6:**
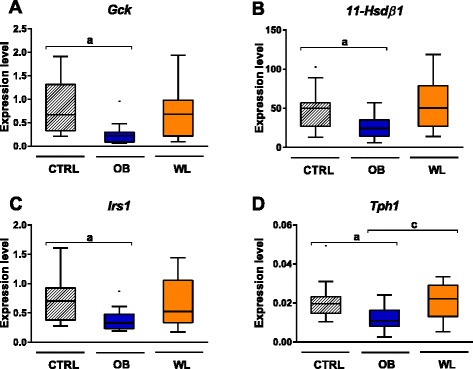
Maternal weight trajectories affect the expression of genes implicated in metabolism. We assessed the expression level of 32 genes implicated in metabolism or development using TaqMan low-density arrays. The expression of *glucokinase* (**a**) in the fetal liver and *hydroxysteroid 11-β dehydrogenase 1* (**b**), *insulin receptor substrate 1* (**c**), and *tryptophan hydroxylase 1* (**d**) in the placental labyrinth was affected by maternal obesity or weight loss at E18.5. (*a*) *P*_adj_ < 0.05 OB vs. CTRL, (*c*) *P*_adj_ < 0.05 WL vs. OB. CTRL (*n* = 16), OB (*n* = 14), WL (*n* = 16)

Among the investigated metabolic genes, only glucokinase (*Gck*) was affected. Its expression was reduced in the liver of OB fetuses compared to that of CTRL and normalized in WL fetuses (Table 1, Fig. 6a).

### Maternal obesity and weight loss altered the expression of epigenetic machinery and metabolic genes in the placenta

**Fig. 7:**
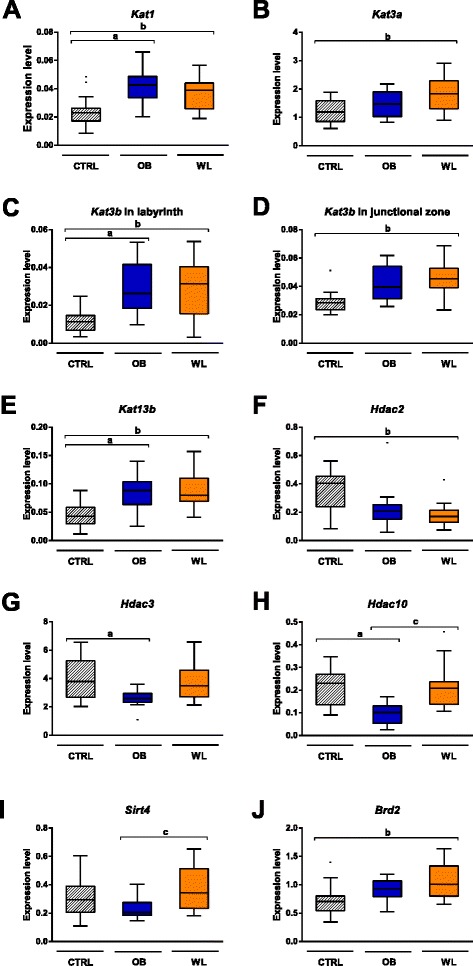
Genes implicated in histone acetylation are differentially expressed in the placenta at E18.5. We assessed the expression level of 60 epigenetic machinery genes using TaqMan low-density arrays. The expression of *Kat1* (**a**), *Kat3a* (**b**), *Kat3b* (**c**), *Kat13b* (**e**), *Hdac2* (**f**), *Hdac3* (**g**), *Hdac10* (**h**), *Sirt4* (**i**), and *Brd2* (**j**) was affected by maternal obesity and weight loss in the labyrinth. Only *Kat3b* (**d**) was differentially expressed in the junctional zone. (*a*) *P*_adj_ < 0.05 OB vs. CTRL, (*b*) *P*_adj_ < 0.05 WL vs. CTRL, (*c*) *P*_adj_ < 0.05 WL vs. OB. CTRL (*n* = 16), OB (*n* = 14), WL (*n* = 16)

Maternal obesity and weight loss altered the expression of 12 epigenetic genes in the placental labyrinth and one gene in junctional zone (Fig. 7, Table 1). Expression of the lysine acetyltransferases *Kat1* (*Hat1*), *Kat3b* (*Ep300*), and *Kat13b* (*Ncoa3*) was higher in the labyrinth of OB dams compared to that of CTRL dams (Fig. 7a, c, e). Expression of the lysine methyltransferase *Kmt1d* (*Ehmt1*) and the histone deacetylases *Hdac3* and *Hdac10* was reduced in the labyrinth of OB dams (Table 1, Fig. 7g, h). In WL dams, responses in the placenta highly differed between the genes. There was no restoration of expression for *Kat1*, *Kat3b*, and *Kat13b*. On the contrary, *Kmt1d*, *Hdac3*, and *Hdac10* expression was restored.

Expression of *Sirtuin 4*, *Mecp2*, and *Mbd5* was higher in WL than in OB labyrinth, but similar to CTRL (Table 1, Fig. 7i). Finally, some genes were not altered by maternal obesity but by maternal weight loss: *Kat3a* and *Brd2* were upregulated and *Hdac2* was downregulated in the labyrinth; *Kat3b* was upregulated in the junctional zone (Fig. 7b, d, f, j). For these four genes, the medians and the distributions in OB group were intermediate between CTRL and WL groups.

We showed that the expression of hydroxysteroid 11-beta dehydrogenase 1 (*Hsd11β1*), insulin receptor substrate 1 (*Irs1*), and tryptophan hydroxylase 1 (*Tph1*) was downregulated in the placental labyrinth of OB dams (Table 1, Fig. 6b–d). There was no difference between WL and CTRL for these metabolic genes. None of the investigated metabolic or developmental genes were affected in the junctional zone.

Thus, the expression of 30 % of epigenetic machinery genes (18 out of 60 studied) was altered by maternal weight trajectories in three tissues. Fifteen percent of genes (9/60) was differentially expressed in the fetal liver, 20 % (12/60) in the labyrinth, and 1.7 % (1/60) in the junctional zone. The histone acetylation pathway (KATs, HDACs, and BRDs) was particularly altered: 78 % of differentially expressed genes in the liver (7/9), 75 % in the labyrinth (9/12), and the only gene in the junctional zone.

Maternal obesity had an important effect on the expression of metabolic and epigenetic genes in the fetal liver and placental labyrinth, but not in the junctional zone. Maternal preconceptional weight loss allowed a global restoration of transcription to CTRL levels. However, the expression of certain genes (for example, *Kat1*, *Kat3b*, and *Kat13b* in the labyrinth and *Brd2* in the liver) was not restored.

## Discussion

**Fig. 8:**
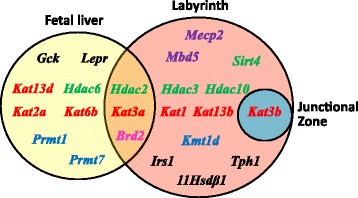
Differentially expressed genes in the fetal liver, placental labyrinth, and junctional zone. *Venn diagram* shows the names of the genes altered by maternal weight trajectories in the three tissues. Metabolic genes are represented in *black*, methyl CpG-binding proteins in *violet*, arginine methyltransferases and lysine methyltransferases in *blue*, histone deacetylases in *green*, lysine acetyltransferases in *red*, and bromodomain-containing proteins in *pink*

In the present study, we showed that maternal chronic obesity lead to FGR at E18.5, while the weight loss induced by a nutritional intervention performed in preconceptional period allowed a partial fetal weight restoration. These phenotypic changes were associated with a transcriptional response in the fetal liver and placental labyrinth: we showed that 23 genes were significantly altered by maternal weight trajectories in at least one of three tissues between two maternal groups (Fig. 8). Our results identified that epigenetic machinery gene expression is clearly sensitive to maternal weight trajectories, especially for genes involved in histone acetylation.

### Maternal obesity-induced FGR is associated with altered expression of epigenetic modifiers in the fetal liver and placental labyrinth

In our model, OB mice were severely obese and presented the characteristics of metabolic syndrome after 4 months of HFD. This resulted in FGR at E18.5, which is consistent with the previous studies in mice [11, 15, 36, 37]. However, in some mouse models, fetuses from OB dams displayed an overgrowth, and it associated with altered placental transport [34, 38]. These discrepancies could be explained by differences in diet composition, especially by high-sugar content. In humans and rats, maternal obesity also associates with an elevated fetal weight [3, 4, 39]. As maternal obesity associates also with FGR in humans, our study provides a mouse model for investigation of the molecular mechanisms of this pathology, which remain unknown [6].

Maternal HFD alters hepatic function and structure in the fetus [14, 15]. In OB dams, we report reduced transcription of *Gck*, the enzyme controlling the synthesis of hepatic glycogen and implicated in type 2 diabetes. *Gck* expression could remain reduced after birth and lead to impaired glucose metabolism [40, 41]. Placental function is also affected by obesity or HFD [11, 12, 34, 38, 42, 43]. In our study, *Hsd11β1*, *Irs1*, and *Tph1* were downregulated in the labyrinth of OB dams, suggesting that alterations of placental function could contribute to FGR. Hydroxysteroid dehydrogenases 11β control the passage of glucocorticoids from the mother to the fetus. In humans, placental expression of *HSD11β1* is associated with birth weight [44]; placental *HSD11βs* expression is reduced in SGA neonates [45]. Impaired placental insulin signaling is associated with obesity, gestational diabetes mellitus, or intrauterine growth restriction (IUGR) [46, 47]. Insulin regulates placental growth, vascularization, glycogen, and lipid storage [48]. Reduced expression of *Irs1* in OB dams could lead to insulin resistance in placental labyrinth and affect these processes. Finally, downregulation of *Tph1* could impair the transformation of maternal tryptophan to serotonin, a process that is necessary for proper fetal brain development [49, 50].

Metabolic diseases are associated with alteration of epigenetic marks [51]. A key finding in this study is that maternal obesity has a major impact on the expression of epigenetic regulators in the fetal liver and placental labyrinth at term. The actors of the histone acetylation pathway were particularly affected. Some families of epigenetic modifiers were not affected by obesity in our study: DNA methyltransferases, TET methylcytosine dioxygenase (TET) proteins, methyl-binding proteins, and lysine demethylases. But this does not preclude changes at previous developmental stages.

Transcript levels of arginine methyltransferases *Prmt1* and *Prmt7* were upregulated in the liver of OB fetuses. Prmts, which catalyze the methylation of arginine histone residues, are implicated in hepatic gluconeogenesis [52]. Prmt1 dimethylates the arginines on forkhead box protein O1 (FOXO1), inducing the translocation of this transcription factor into the nucleus and activation of its target metabolic genes [53]. No implication of Prmt7 in hepatic metabolism or development is known. Implications of the histone arginine methyltransferase activity in FGR or in response to maternal nutritional environment remain to be determined. Only one of the seven studied lysine methyltransferases was differentially expressed in our study: *Kmt1d* was downregulated in the labyrinth of OB dams. No implication of this lysine methyltransferase in placental biology has yet been reported.

### Preconceptional weight loss is beneficial to fetal growth and induces an adaptation at the transcriptional level

To our knowledge, it is the first mouse model to study the effects of preconceptional weight loss induced by nutritional intervention on fetal growth and on the transcriptional response in fetal and placental tissues. Despite the normalization of maternal phenotype at conception (Fig. 2), fetuses of WL dams presented transcription differences compared with fetuses of CTRL dams.

The nutritional intervention and preconceptional weight loss restored the FGR observed in OB fetuses. Interestingly, the proportion of SGA fetuses was comparable to the proportion of SGA fetuses from CTRL dams. The first human cohort studies point at rather beneficial effects of maternal weight loss on fetal outcomes, namely a lower risk of large for gestational age (LGA) infants compared to obese patients [27, 28, 54]. In sheep, maternal obesity induced macrosomia and elevated heart, liver, and perirenal adipose tissue weights. A nutritional intervention normalized the fetal and organ weights [55]. In rat studies, there was no effect of maternal obesity or weight loss on fetal weight [56, 57]. Taking into account our results, preconceptional weight loss appears beneficial for fetal growth, counteracting the adverse effects of maternal obesity.

In our expression study, we observed different profiles of transcriptional response to maternal weight loss: mRNA levels of a subset of genes were completely or partially normalized, while other genes remained altered. Overall, the placenta and fetal liver in WL group presented an adaptation in gene expression with high individual variability for some genes.

Finally, some genes were differentially expressed only in WL group: the methyl-DNA binding proteins *Mecp2* and *Mbd5* were upregulated in the labyrinth of WL compared with CTRL and OB. These DNA methylation “readers” are implicated in obesity (MeCP2) and glucose homeostasis regulation (MBD5) [58–60]. In the placenta of calorie-restricted mice, MeCP2 binding to hypermethylated CpG island of *Glut3* was enhanced [61].

We observed a restoration of *Gck* mRNA levels in the fetal liver of WL offspring, and a restoration of *Hsd11β1*, *Irs1*, and *Tph1* levels in the placental labyrinth. Similar results were obtained for *Hsd11β1* in a sheep model of maternal obesity and nutritional preconceptional intervention [62]. The normalization of expression of these genes involved in glucose, glucocorticoid and serotonin metabolism, and insulin signaling by maternal weight loss could abolish the negative effects induced by maternal obesity.

In this study, maternal obesity, despite the nutritional intervention prior to conception, was retained in the gene expression pattern. WL females were severely obese throughout their puberty, when HFD consumption and high adiposity could have affected their gonads. Obesity causes lipid accumulation, mitochondrial dysfunction, and chromosomal abnormalities in oocytes, as well as a modification of follicular liquid content [63, 64]. This can induce FGR and developmental defects [65]. The negative consequences of maternal obesity could be transmitted, at least in part, via alteration of oocyte function and epigenome. Methylation levels of genes involved in the lipid metabolism could be transmitted to the blastocyst and to adult progeny in mice [66]. In another study, maternal HFD and obesity induces hypermethylation of *Lep* and hypomethylation of *Pparα* in the oocytes and in the liver of adult offspring [67]. The oocyte epigenetic machinery and acetylation levels are sensitive to maternal diabetes [68]. Therefore, in our study, despite the normalization of metabolism by nutritional intervention, some epigenetic marks affected by obesity could be retained in the WL group. Weight loss could also trigger the addition of novel epigenetic marks in oocytes. These epigenetic alterations could provide a mechanism that explains the “memorization” of an obesogenic environment.

### The balance between “writers” and “erasers” of histone acetylation could play a role in obesity-induced FGR

In our expression profiling, we studied the transcription of 29 genes involved in histone acetylation (KATs, HDACs, and BRDs), out of 60 epigenetic genes (48 %). Remarkably, 78 % of differentially expressed epigenetic genes in the fetal liver (7 out of 9) and 75 % in the labyrinth (9 out of 12) are involved in histone acetylation. The only differentially expressed gene in the junctional zone (*Kat3b*) is also involved in acetylation pathway. The three genes in common between the liver and labyrinth and the gene differentially expressed in the two placental layers are involved in histone acetylation. Seven members of KAT family, “writers” of lysine acetylation, were upregulated in the fetal liver and placental labyrinth of OB dams. Interestingly, their expression was restored in the fetal liver (*Kat2a*, *Kat3a*, *Kat6b*, and *Kat13d*) of WL dams, but there was clearly no restoration in the labyrinth (*Kat1*, *Kat3a*, *Kat3b*, and *Kat13b*). Moreover, the expression of the majority of HDACs (*Hdac2*, *Hdac3*, and *Hdac10*) that mediate the opposite reaction was reduced in the fetal liver and placental labyrinth of OB dams.

Therefore, we observed a disruption in the balance between the expression of “writers” and “erasers” of lysine acetylation in offspring from obese mother at term of gestation (Table 2). This could lead to an increased level of histone acetylation in FGR offspring of OB dams. In primates, global levels of histone 3 (H3)K14ac were upregulated in the liver at term and after birth in the case of chronic maternal HFD consumption [69]. This hyperacetylation is consistent with reduced expression and enzymatic activity of Hdac1 and Sirt1 [70]. In mice, maternal HFD increased the level of H3K14ac in the fetal liver at E18.5, and this effect persisted after birth [71]. Interestingly, expression of *Hdac1*, *Hdac3*, and *Sirt1* was unaltered at the same stage. In rats, the *Pepck* gene implicated in gluconeogenesis is enriched in H4ac in the liver of fetuses from dams on a HFD, which is consistent with its high expression and fetal hyperglycaemia [39]. In diabetic mice, the expression of *KATs* was upregulated in oocytes, while the expression of *HDACs* was downregulated, as in our study, and the dynamics of acetylation at different lysine residues was disturbed during oocyte maturation [68].

**Table 2:** Maternal weight trajectories affect the expression of genes involved in histone acetylation

	Writers: KATs		Erasers: HDACs		Readers: Brd2	
	OB	WL	OB	WL	OB	WL
Fetal liver	↑	Restoration	↑ or ↓	No restoration (trend)/restoration	↑	No restoration
Labyrinth	↑	No restoration	↓	No restoration/restoration	**=**	↑
Junctional zone	**=**	↑	-	-	-	-

↑ upregulation, ↓ downregulation, **=** no significant difference, - no differential expression in this tissue, *KAT* lysine acetyltransferases, *HDAC* histone deacetylases, *BRD* bromodomain-containing proteins

These studies mainly tested the expression of acetylation-related genes. One purpose of our study was to analyze the expression of different families of epigenetic modifiers. The only other extensive report focused on 67 epigenetic modifiers in transcriptomic data from fetal lung, liver, kidney, heart, and placenta of IUGR fetuses, induced by low-protein maternal diet in rats [72]. Histone acetylation modifiers were unchanged while DNA methyltransferases (DNMTs) were differentially expressed in the fetal liver and HDACs in the lung. Therefore, maternal undernutrition or overnutrition could affect different biological processes yet both resulting in FGR.

Some of the differentially expressed KATs or HDACs revealed in our study are implicated in metabolic processes and associated with metabolic diseases, according to different studies in mice and humans. *Hdac2* and *Hdac6* are involved in adipogenesis [73]. HDAC3 is an important regulator of hepatic lipid metabolism in a circadian manner and invalidation of its gene induces hepatic steatosis [74]. Single nucleotide polymorphisms in *KAT13D* (*CLOCK*) gene are associated with obesity and weight loss success in humans [75, 76]. CLOCK is a major circadian regulator: in mice, its mutation disrupts the circadian rhythms and induces a metabolic syndrome [77]. *Kat13b* is involved in energy expenditure and therefore obesity [78]. *Kat3a* is implicated in diabetes by acting on the transcription of gluconeogenetic genes [79]. Kat3b regulates the expression of genes involved in lipogenesis and gluconeogenesis in the liver [80]. Thus, KATs and HDACs have important roles in metabolism and their alteration in obese offspring could indicate major changes in placental and hepatic function.

Bromodomain proteins recognize acetylated lysines and recruit other enzymes to form a multiprotein complex that further enhances transcriptional activity [81]. Brd2 is a negative regulator of adipogenesis via transcriptional repression of *Pparγ.* Heterozygous mice invalidated for *Brd2* are obese and have surprisingly better glucose tolerance [82]. The meaning for the increased *Brd2* expression in OB group observed in our study (Table 2, Fig. 5) remains to be determined.

Overall, our results show that epigenetic machinery genes are sensitive to maternal environment and that histone acetylation pathway is particularly affected. Epigenetic modifiers should be considered when studying the offspring’s response to maternal metabolic disturbances.

### Limitations and strength of the study

In this study, we used a mouse model of obesity-induced FGR, relevant for the investigation of epigenetic mechanisms underlying this pathology, in relation to feto-placental development. This is also the first report of the effects of preconceptional maternal weight loss induced by nutritional intervention on feto-placental growth and gene expression at term. This important contribution highlights the consequences of maternal body weight changes on fetal outcomes and molecular processes, providing evidence to the elaboration the preconceptional counseling for obese women. In our nutritional protocol, we used well-controlled purified HFD containing 59.9 % calories from lipids (mainly saturated and monounsaturated fatty acids). This diet is largely used in metabolic studies in rodents and is different from the “Western diet” characterized by a high-lipid and high-sucrose content [36, 38]. Dietary lipids can have a direct impact on gene transcription by regulation of transcription factors and epigenetic enzymes [24, 83]. However, it is not possible to distinguish the proper effects of maternal obesity (hormonal and inflammatory factors, insulin resistance, and hyperglycemia) and that of dietary lipids in our study. Others tried to address this issue showing differential effects of maternal adiposity and HFD on offspring phenotype, but the effects of lipids on epigenetic processes during early development need further investigation [84, 85]. Another important point to notice is that in our model, 7 weeks of mating were necessary to obtain a large enough sample size. We therefore included maternal age/diet duration (indistinguishable according to our protocol) in our statistical model. Interestingly, the proportion of variance in fetal weight and FPI explained by the maternal age are comparable to the proportion of variance explained by group. This highlights the relevance of maternal age/diet duration for fetal weight and the importance to include it as a covariate.

In our expressional screening, we used a high-output reverse transcription (RT)-real-time polymerase chain reaction (qPCR) technique that simultaneously assessed 96 transcripts. We have studied 60 epigenetic machinery genes, which gives a large overview of the maternal impact on epigenetic regulation processes. These genes were chosen based on their implication in metabolic processes or on our previous study where the effect of maternal HFD during pregnancy showed an altered expression of seven epigenetic genes [30]. Our goal was to study different families of epigenetic modifiers (DNMTs, TETs, lysine methyltransferases (KMTs), lysine-specific demethylases (KDMs), KATs, HDACs, BRDs) because the literature was particularly focused on histone acetylation and DNA methylation. However, some interesting epigenetic families were not taken into account, like the gene-encoding proteins from the polycomb or tritorax families, or nucleosome remodelers, such as switch/sucrose non-fermentable (SWI/SNF). The expression of these genes has never been studied in the context of obesity. Moreover, the expression changes reported here are transcript levels. There may be differences between mRNA levels and proteins level or enzymatic activity. In other studies, Hdac1 and Sirt1 mRNA and protein levels were both affected in the same direction in the context of maternal obesity or HFD [69, 70].

The originality of our study was to compare different parts of the placenta—labyrinth and junctional zone—which have different structures, cellular content, and function. Indeed, we observed a striking difference in terms of gene expression in these two placental layers, highlighting that obesity could have plausible different impacts on them. We observed a smaller transcriptional response in junctional zone in comparison with labyrinth. A plausible explanation could be that the labyrinth, due to its intimate contact with maternal blood, is more reactive at the transcriptional level, in order to adapt to a dynamic environment. Interestingly, the only affected gene in the junctional zone (*Kat3b*) is differentially expressed in a similar pattern in the labyrinth. KAT3b is implicated in preeclampsia, a disease associated with placental insufficient vascularization, and with the regulation of placental *Hsd11β2* expression via elevated H3K9ac and H3K27ac in humans [86, 87]. Our study highlights a potential important role of KAT3b in the placenta in response to maternal metabolism. Its epigenetic targets need further investigation.

## Conclusions

In our mouse model, maternal obesity induced FGR and reduced placental efficiency at term. These phenotypic changes were associated with alterations of the expression of genes involved in epigenetic processes in the fetal liver and placenta. Nutritional intervention during the preconceptional period allowed maternal weight loss and the normalization of metabolic parameters at mating. In the offspring of WL mothers, fetal growth was partially restored and the transcription was normalized only for a subset of genes affected by maternal obesity. Thus, the history of maternal obesity has an impact on fetal growth and transcriptional activity. The epigenetic machinery is highly sensitive to maternal weight trajectories, which could lead to an altered epigenome in the offspring. Histone acetylation modifiers represented a major part of the differentially expressed genes in OB and WL groups that could account for reminiscence of the obese status.

This study highlights the importance of investigating the mechanisms of regulation of histone marks in response to environmental insults. The link between histone modifiers, histone acetylation levels, and placental and hepatic function should be established. Alteration of the epigenome early during ontogenesis, could be a mechanism of “memorization” of the environment in utero, contributing to particular gene expression patterns and thus to adult phenotype establishment. It could be an underlying mechanism explaining the conditioning of the offspring health later in life [9, 88]. Advances in this direction should help to unravel the molecular mechanisms of developmental conditioning induced by maternal weight trajectories.

## Methods

### Animal experiments

The COMETHEA ethical committee (Comité d’éthique pour l’expérimentation animale), registered with the national Comité National de Réflexion Ethique sur l’Expérimentation Animale under the no. 45 approved this protocol (visa 12/062). Four-week-old female and 7-week-old male C57Bl/6J OlaHsd mice were received from Harlan Laboratory (Venray, Netherlands) and housed in Unité d'Infectologie Expérimentale des Rongeurs et Poissons (IERP; INRA, Jouy-en-Josas, France). After 1 week of adaptation, the mice were placed in individual cages at controlled temperature (22 ± 2 °C) with a 12-h-light/12-h-dark cycle. Mice had ad libitum access to water and food, and paper towel was provided for nest building.

Five-week-old control females (CTRL) were fed a control diet (CD, 10 % from fat, 70 % from carbohydrates, 20 % from protein; #D12450K) and obese females (OB) were fed a high-fat diet (HFD, 59.9 % from fat, 20.1 % from carbohydrates, 20 % from protein; #D12492) during 4 months before conception and throughout gestation (Fig. 1). Diets were purchased in pellet form from Research Diets (New Brunswick, NJ, USA). After 2 months of HFD (at 14 weeks of age), a subset of the OB females was placed on a CD in order to induce a weight loss (WL group) for the remaining 2 months before conception. Overall, 20 CTRL, 23 OB, and 19 WL females followed nutritional protocol. Females and food on the grid were weighed twice a week. Food intake (FI) was calculated as (food day *n* (g) − food day 0 (g))/*n* days, with 3.85 kcal/g for CD and 5.24 kcal/g for HFD. FI-to-body-weight ratio (kcal/kg of body weight/day) was calculated for each mouse. Measurement of fasting cholesterolemia, glycaemia, and oral glucose tolerance test (OGTT) were performed at age 13 and 22 weeks (following 2 and 4 months of diet).

Females were mated individually with randomly assigned, chow-fed (Special Diets Services, Witham, Essex, England; #801030 RM3A) 8–9-week-old males (*n* = 12). The presence of vaginal plug represented embryonic day 0.5 (E0.5). If no plug was observed, females were mated with another male according to their oestrus cycle. All females were mated between 23 and 30 weeks of age and respective diets were maintained throughout mating period and gestation. Body weight of pregnant females was recorded at E0.5 and E18.5. Gestational weight gain was calculated as (body weight at E18.5 (g) − body weight at E0.5 (g))/body weight at E0.5 (g). Pregnant CTRL (*n* = 14), OB (*n* = 11), and WL (*n* = 17) females were weighed and sacrificed by cervical dislocation at E18.5. Tissue sampling was performed on a table maintained at 4 °C. Fetuses and placentas were removed from the uterine horn and placed in a solution of PBS 1×. Maternal carcasses (bodies without the uterus, fetuses, and placentas) were weighed, and proper maternal body weight gain was calculated as (carcass weight at sacrifice (g) − body weight at E0.5 (g))/body weight at E0.5 (g). Fetal development stage was in accordance with the “Theiler Staging Criteria for Mouse Embryo Development” (TS 26). One CTRL fetus and one OB fetus were removed from analysis and tissue collection because of placental necrosis associated with weak fetal weight. Fetal sex was determined by visual examination of the gonads. Fetuses and placentas were weighed (36 CTRL females (F) and 39 CTRL males (M); 35 OB F and 39 OB M; 61 WL F and 58 WL M). Maternal group did not alter the sex ratios of the litters (proportion test: *p* = 0.81; CTRL 48 % F, 52 % M; OB 47 % F, 53 % M; WL 51 % F, 49 % M). The placental labyrinth and junctional zone were separated and collected, along with the fetal liver. Tissue samples were snap-frozen in liquid nitrogen and stored at −80 °C.

### Experimental procedures

Fasting blood glucose levels were measured in all females (20 CTRL, 23 OB, 19 WL). OGTTs were performed in a subset of females on a HFD, which showed a maximal body weight and weight gain (*n* = 11–12 per group) and in CTRL females within an average range of weight. After 6 h of fasting (8:00 a.m. to 2:00 p.m.), a bolus of glucose (2 g/kg of body weight) was delivered into the stomach of conscious mice with a gavage needle. Tail vein blood glycaemia was measured in duplicates using an Accu-Chek Performa blood glucose meter (Roche diagnostics GmbH, Germany) at time 0, 20, 40, 60, 90, and 120 min. This protocol was described as the best discriminating glucose tolerance between HFD vs. CD mice [89].

Submandibular vein blood (300 μl) from conscious mice was collected in heparinized tubes (Choay heparin, Sanofi-aventis, Paris, France) after 6 h of fasting. Blood samples were centrifuged for 10 min at 1500*g* at 20 °C. Plasma was collected and stored at −20 °C. Total cholesterol plasma levels were measured by colorimetric dosage on Vitros system in Ambroise Paré Hospital (Boulogne-Billancourt, France).

### Statistical analysis of physiological data

All analyses were performed with the R statistical software. For the entire preconceptional period, analyses were performed on all mice (20 CTRL, 23 OB, 19 WL). After mating, analyses were restricted to pregnant females (14 CTRL, 11 OB, 17 WL) and their litters. Linear mixed models were used to model the evolution of parameters such as weight, food intake, and food efficiency with time, using the lmer function of the lme4 package in R [90]. Estimates of the slopes for each parameter were reported as “*β*.” Reported *p* values were obtained with a likelihood ratio test, thanks to the lrtest function of the lmtest package [91]. ANOVA was used to test differences at each time point, thanks to the aov function. Reported *p* values were obtained with a likelihood ratio test, thanks to the lrtest function of the lmtest package [91]. We performed complete case analyses (i.e., we removed all missing data for each individual, none were imputed). All *p* values were adjusted for multiple comparisons using the p.adjust function with BH correction [92]. If significance was found, Tukey’s post hoc test was used to determine which groups differ from one another. Effect size was reported as the proportion *R*^2^ of the variance in the variable of interest (e.g., body weight, cholesterol, fetal weight) that is explained by maternal group, among the variance not already explained by the covariates. For most variables of interest, groups showed unequal variances. Therefore, investigated parameters were Box-Cox transformed, using the powerTransform and bcPower functions of the car package [93]. This function estimates a transformation for the variable *z* from the family of transformations indexed by the parameter lambda that makes the residuals from the regression of the transformed *z* on the predictors as close to normally distributed as possible. We used Pearson’s product moment correlation coefficient to test a correlation between fetal weight and placental weight. To adjust for factor “mother,” we conducted a regression of fetal weight on mother and a regression of placental weight on mother. The correlation test was performed on the residuals of both models. We used a proportion test to determine differences in sex ratios in the offspring. We calculated a proportion of SGA fetuses as described previously, using *Z*-score of 1.28 [94]. Logistic regression was used to see the impact of maternal group on the odds of being SGA, using the SGA status of the fetus as the response variable (0 = not SGA and 1 = SGA) and age, sex, and litter size as explanatory variables. Data are represented as Tukey’s boxplots indicating the median, 25th, and 75th percentiles. Whiskers indicate the 5th and 95th percentiles. Outliers are shown as dots.

### Expression analysis

#### RNA extraction and DNAse treatment

Only litters with 6–9 fetuses were included in the expression analysis. For each fetus, total RNA was extracted from 20 to 50 mg of the placental labyrinth, junctional zone, or fetal liver. Tissue was reduced to a powder in liquid nitrogen and homogenized in 500 μl of TRIZOL reagent in Mixer Mill MM300 (Qiagen) with one tungsten ball for 2 min at 20 Hz twice. Then, RNA was extracted according to the manufacturer’s instructions for RNA isolation (Life Technologies). The aqueous phase containing RNA was collected using Phase Lock Gel Heavy tubes (5 Prime, Hamburg, Germany). The extracted RNA was resuspended in 100 μl of RNase-free water and stored at −20 °C. RNA concentration and purity (A260/A280) was measured using NanoDrop spectrophotometer (NanoDrop Technologies), and a DNase treatment was performed (DNA-free kit, AM1906, Ambion, Life Technologies). The quality of RNA samples was assessed using Bio-Analyzer Agilent 2100 (Agilent Technologies). The RNA integrity number (RIN) of all samples was between the range of 8.8 and 10. For each tissue, 2.5 μg of RNA per fetal sample was pooled according to litter, sex, and maternal diet (*n* = 8 CTRL F, 8 CTRL M, 7 OB F, 7 OB M, 8 WL F, 8 WL M).

#### Reverse transcription

Six hundred nanograms of RNA was converted into cDNA using the High Capacity cDNA Reverse Transcription Kit with RNase Inhibitor (Applied Biosystems) according to the manufacturer’s recommendations. Reverse transcription were performed in duplicates that were pooled and stored at +4 °C.

#### Quantitative real-time PCR

Gene expression was quantified using custom TaqMan low-density arrays (TLDAs) (Applied Biosystems). Each array consisted of a 384-well microfluidic card preloaded with primer sets and 6-FAM-labeled TaqMan probes. The format we chose (96a; Cat. No. 4342261) contained four samples per card; for each sample, the expression of 96 genes was measured, including four control assays. The first set of 60 target genes included genes implicated in epigenetic processes and the second set of 32 target genes included genes implicated in placental/hepatic development or energetic metabolism (Additional file 3). We studied the expression of five DNA methyltransferases (DNMTs), 18 histone deacetylases (HDACs), seven KMTs, seven KDMs, nine lysine acetyltransferases (KATs), five methyl-binding domain proteins (MBDs), two bromodomain proteins (BRDs), four protein arginine n-methyltransferases (PRMTs), and three enzymes of DNA hydroxymethylation (TETs). All assays and their assay ID numbers are listed in Additional file 2: Table S1. All probes spanned a gene, an exon-exon junction, except for the following assays: Actb (Mm00607939_s1), Mrpl32 (Mm00777741_sH), Bdnf (Mm04230607_s1), Cebpα (Mm00514283_s1), and Cebpβ (Mm00843434_s1), where primers and probes mapped within a single exon. In addition, the following assays may detect genomic DNA: 18S (Hs99999901_s1), Hdac1 (Mm02391771_g1), Hdac10 (Mm01308118_g1), and Mecp2 (Mm01193537_g1). Thus, all samples were treated with DNase. The experiment was performed on the BRIDGE-ICE platform (INRA, Jouy-en-Josas, France) according to the manufacturer’s instructions. Four samples were run on each TLDA card in simplicates. Each sample reservoir on the card was loaded with 100 μl of the reaction mix: cDNA template (600 ng) mixed with TaqMan Gene Expression Master Mix (Applied Biosystems). After centrifugation (twice 1 min at 1200 rpm, Heraeus Multifuge 3S Centrifuge), the wells were sealed with a TLDA Sealer (Applied Biosystems). Real-time PCR amplification was performed on the 7900HT Real-Time PCR System (Applied Biosystems) using SDS 2.4 software with standard conditions: 2 min 50 °C, 10 min 94.5 °C, 30 s 97 °C (40 cycles), 1 min 59.7 °C.

#### Normalization of expression level

Six potential reference genes were tested on the fetal liver and placental tissue samples: *Eif4a2* (eukaryotic translation initiation factor 4A2), *ActB* (Actin beta), *Tbp* (TATA box-binding protein), *Gapdh* (glyceraldehyde-3-phosphate dehydrogenase), *Mrpl32* (mitochondrial ribosomal protein L32), and *Sdha* (succinate dehydrogenase complex, subunit A, flavoprotein). Using the GeNorm software, *ActB*, *Sdha*, and *Mrpl32* were chosen for normalization of expression levels [95]. Threshold cycle (Ct) values were calculated with the ExpressionSuite v1.0.3 software (Applied Biosystems). The detection threshold was set manually for all genes and was the same for each assay in all tissues. Ct = 39 was used as the cutoff: above this value, expression level was set to 0. Genes were assigned as non-amplified (NA) if more than 15 % of samples were NA. All NA genes (Additional file 4: Table S2; 12 in the fetal liver, 6 in the placental labyrinth, and 3 in the junctional zone) were removed from the analysis. Normalization was performed independently for same-sex samples within each maternal group (CTRL F, CTRL M, OB F, OB M, WL F, WL M). For each of these groups, Ct_[ref]_ was the mean of the three Ct values of the reference genes. Then, expression level of target genes was calculated as 2^−(Ct[target gene] − Ct[ref])^, as previously described [96].

#### Hierarchical clustering

For each tissue, transcription values of each target gene were averaged across same-sex samples within each maternal group (CTRL F, CTRL M, OB F, OB M, WL F, WL M). Only genes with expression >0 were taken into account (80 in the liver, 86 in the labyrinth, and 89 in the junctional zone). Missing Ct values were imputed (7 in the liver, 7 in the labyrinth, and 4 in the junctional zone out of 4416 values per tissue), assigning the mean Ct value of the samples with similar sex and maternal diet. Then, hierarchical clustering was performed using Pearson’s correlation coefficient as distance function and Ward as linkage method.

#### Statistical analysis of TLDA expression study

Analysis was performed with the R statistical software. For each gene, we compared the expression values between males and females within each maternal group. As we did not detect an effect of sex on the expression for any of the studied genes (except for *Kdm5d* and *Uty*, both located on Y chromosome), we pooled males and females within each maternal group. Pairwise comparisons of maternal groups were conducted using a permutation test, as implemented in the oneway_test function of the *coin* package in R [97]. For each set of tests (i.e., all tested genes for a given pair of maternal groups), *p* values were adjusted for multiple testing as proposed by Benjamini and Hochberg [92]. Differences were considered significant when *P*_adj_ < 0.05. Data are shown as means ± St. Dev or as Tukey’s boxplots.

## Additional files

Additional file 1:
**Supplementary Figures S1 to S6.** Food intake (FI) to body weight (BW) ratio in females during the preconceptional period. (a) *P* < 0.05 OB vs. CTRL, (b) *P* < 0.05 WL vs. CTRL, (c) *P* < 0.05 WL vs*.* OB. *n* = 18–20 CTRL, 23 OB, 17–19 WL. **Figure S2.** Fetal weight as a function of litter size in dams at E18.5. Both sexes were combined as there was no effect of sex on fetal weight. **Figure S3.** Placental weight as a function of litter size and sex at E18.5. **Figure S4.** Effect of maternal age on fetal parameters. (A) Relationship between maternal age and fetal weight. (B) Relationship between maternal age and fetal-weight-to-placental-weight ratio index (FPI). For statistical analysis, see text. **Figure S5.** Distribution of fetal weight in CTRL, OB, and WL dams at E18.5. CTRL dams are represented in black, WL dams in brown, and OB dams in blue. The red line represents the 10th percentile of CTRL population. **Figure S6.** Relationship between fetal and placental weight in female and male offspring at E18.5. For statistical analysis, see text and “Methods” section. M: males, F: females. Additional file 2:
**List of the gene symbol and name, and the design strand and TaqMan® Gene Expression Assay IDentification reference for the 96 genes studied on the Custom TLDA.**
Additional file 3:
**Description of the selection criteria of genes for the custom TLDA design and related bibliographic references.**
Additional file 4:
**Expression level and adjusted** ***P*** **values of 92 genes in the fetal liver, placental labyrinth, and junctional zone.** We assessed the expression level of 60 epigenetic machinery genes and 32 genes implicated in metabolism or development in CTRL, OB, and WL females at E18.5 using TaqMan low-density arrays. Data are represented as mean expression levels ± St.Dev. *—differentially expressed genes. NA—non-amplified. Significant differences (*P*_adj_ < 0.05) are indicated in red.

## Abbreviations

BRDs: bromodomain proteins
CD: control diet
CTRL: control group
DNMTs: DNA methyltransferases
DOHaD: developmental origins of health and disease
E18.5: embryonic day 18.5
FGR: fetal growth restriction
FPI: fetal-weight-to-placental-weight ratio index
H3: histone 3
HDACs: histone deacetylases
HFD: high-fat diet
KATs: lysine acetyltransferases
IUGR: intrauterine growth restriction
KDMs: lysine-specific demethylases
KMTs: lysine methyltransferases
LGA: large for gestational age
MBDs: methyl-binding domain proteins
NA: non-amplified
OB: obese group
OGTT: oral glucose tolerance test
PRMTs: protein arginine n-methyltransferases
*P*_adj_: adjusted *p* value
qPCR: real-time polymerase chain reaction
RT: reverse transcription
SGA: small for gestational age
TETs: TET methylcytosine dioxygenases
TLDA: TaqMan low-density array
WL: weight loss group
