# Supplementary figures and images for: Expression of epigenetic machinery genes is sensitive to maternal obesity and weight loss in relation to fetal growth in mice
Source: Clin Epigenetics. 2016 Feb 27;8:22. doi: 10.1186/s13148-016-0188-3 (PMC4769534; doi:10.1186/s13148-016-0188-3)

Figure S1

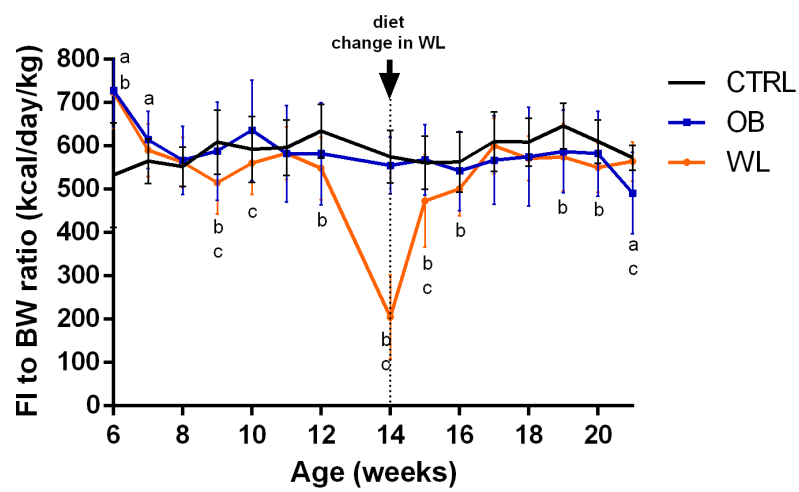

Figure S2.

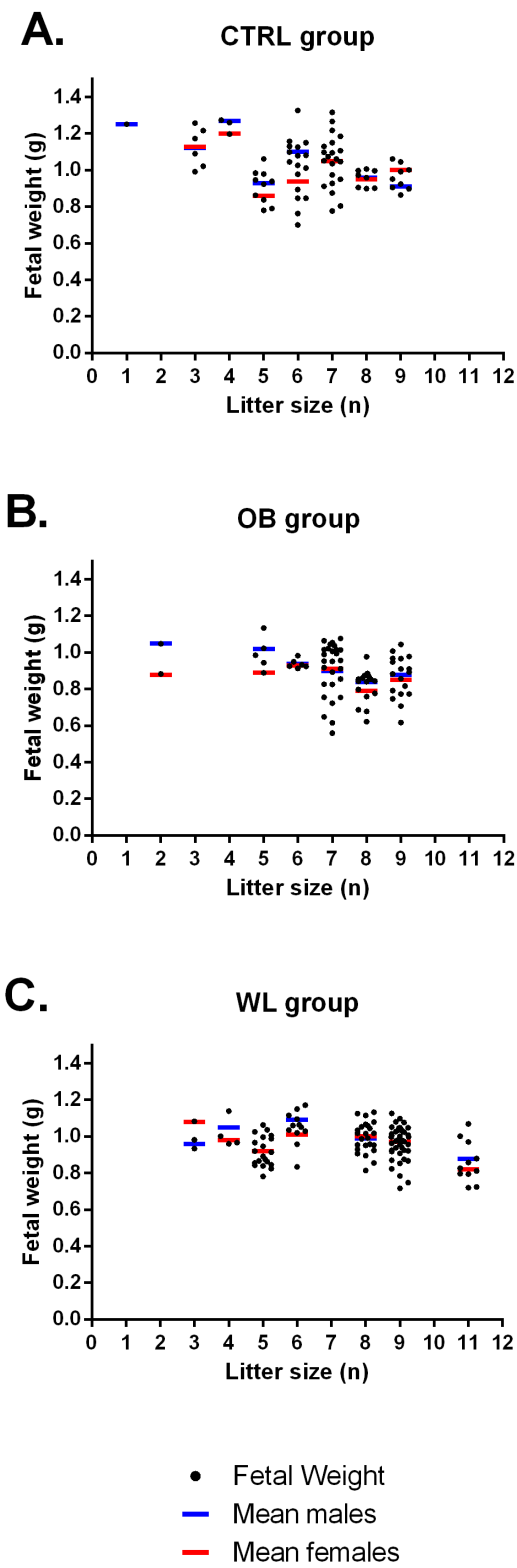

Figure S3.

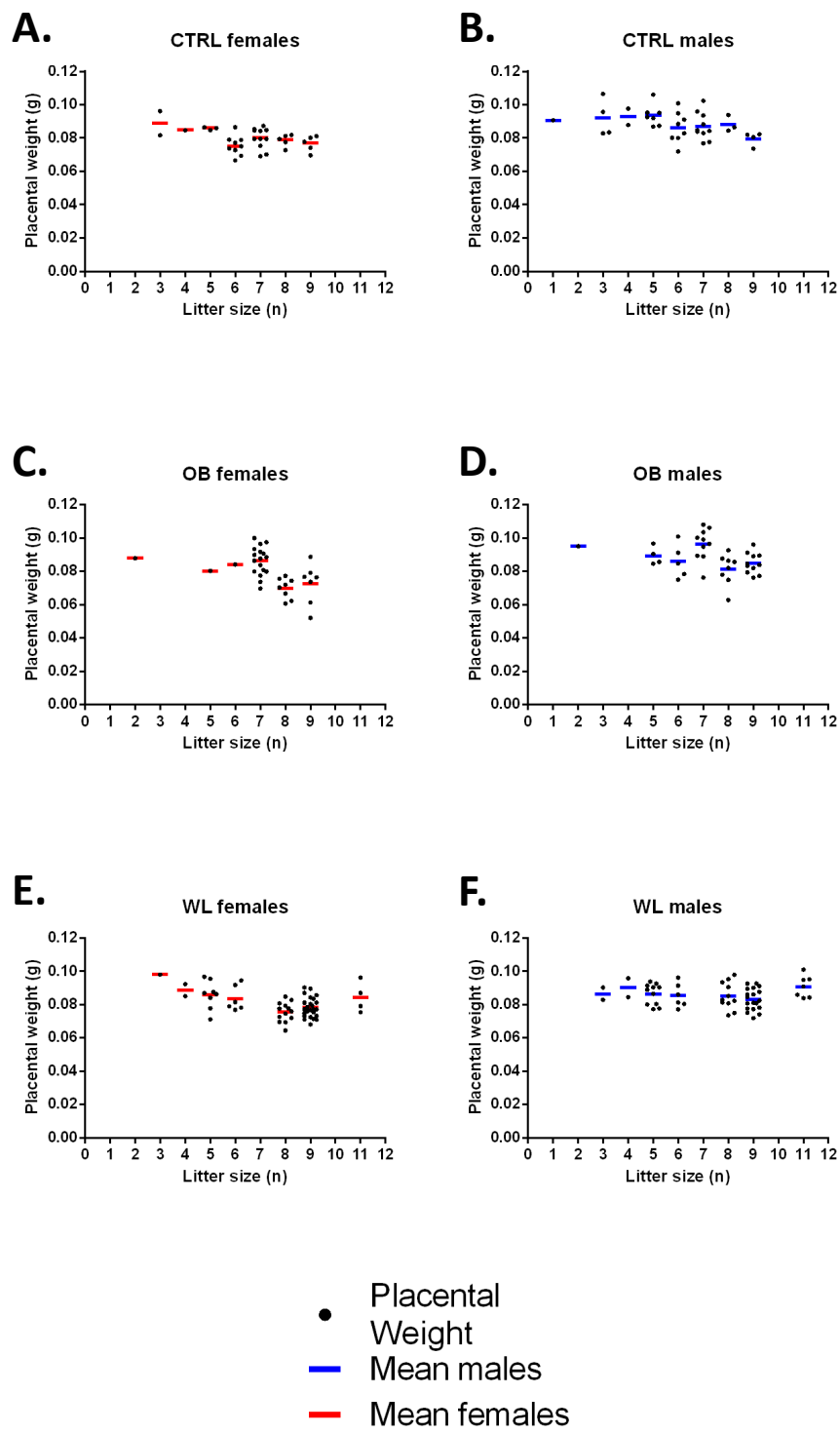

Figure S4.

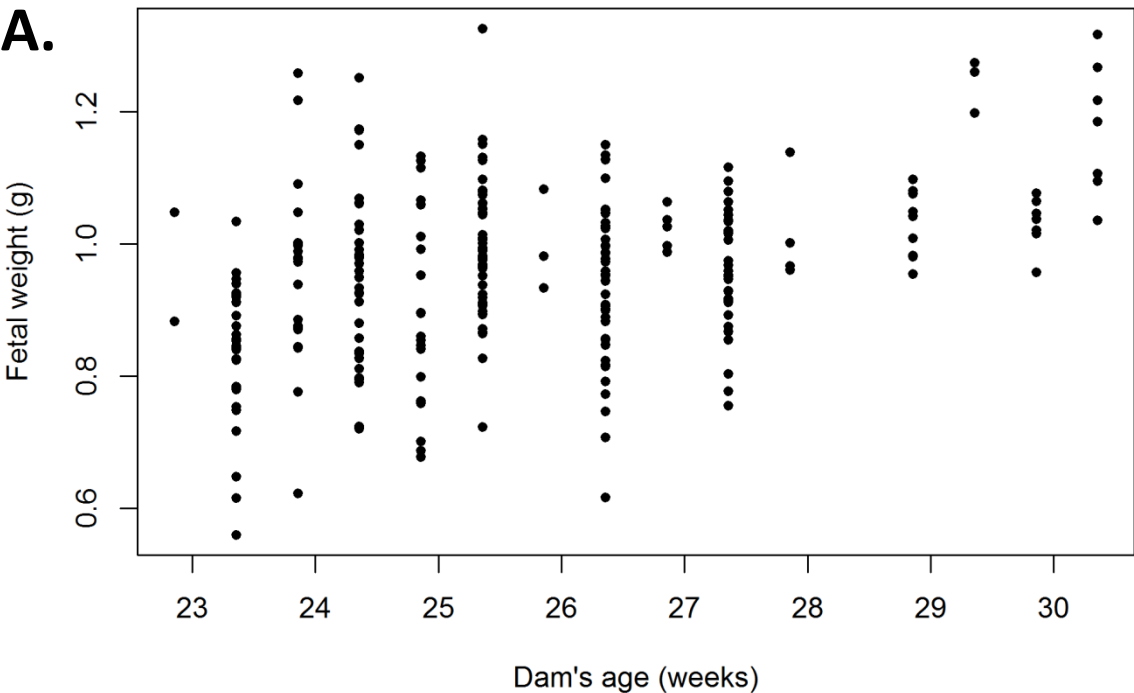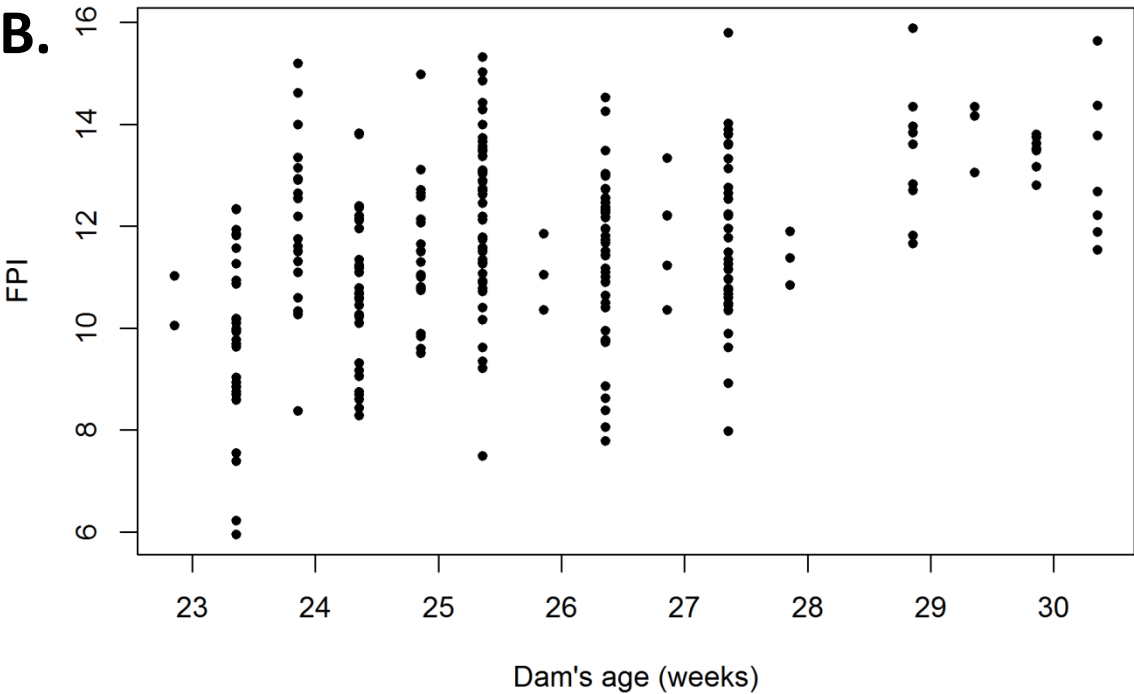

Figure S5.

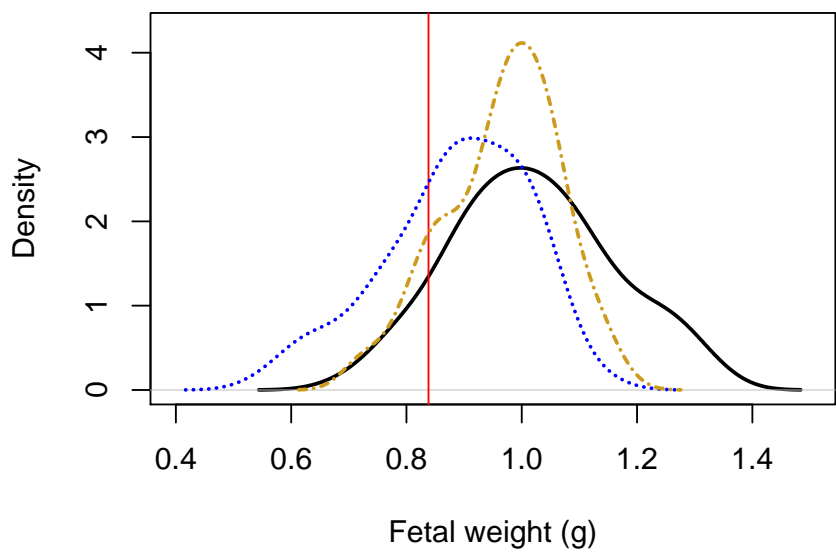

Figure S6.

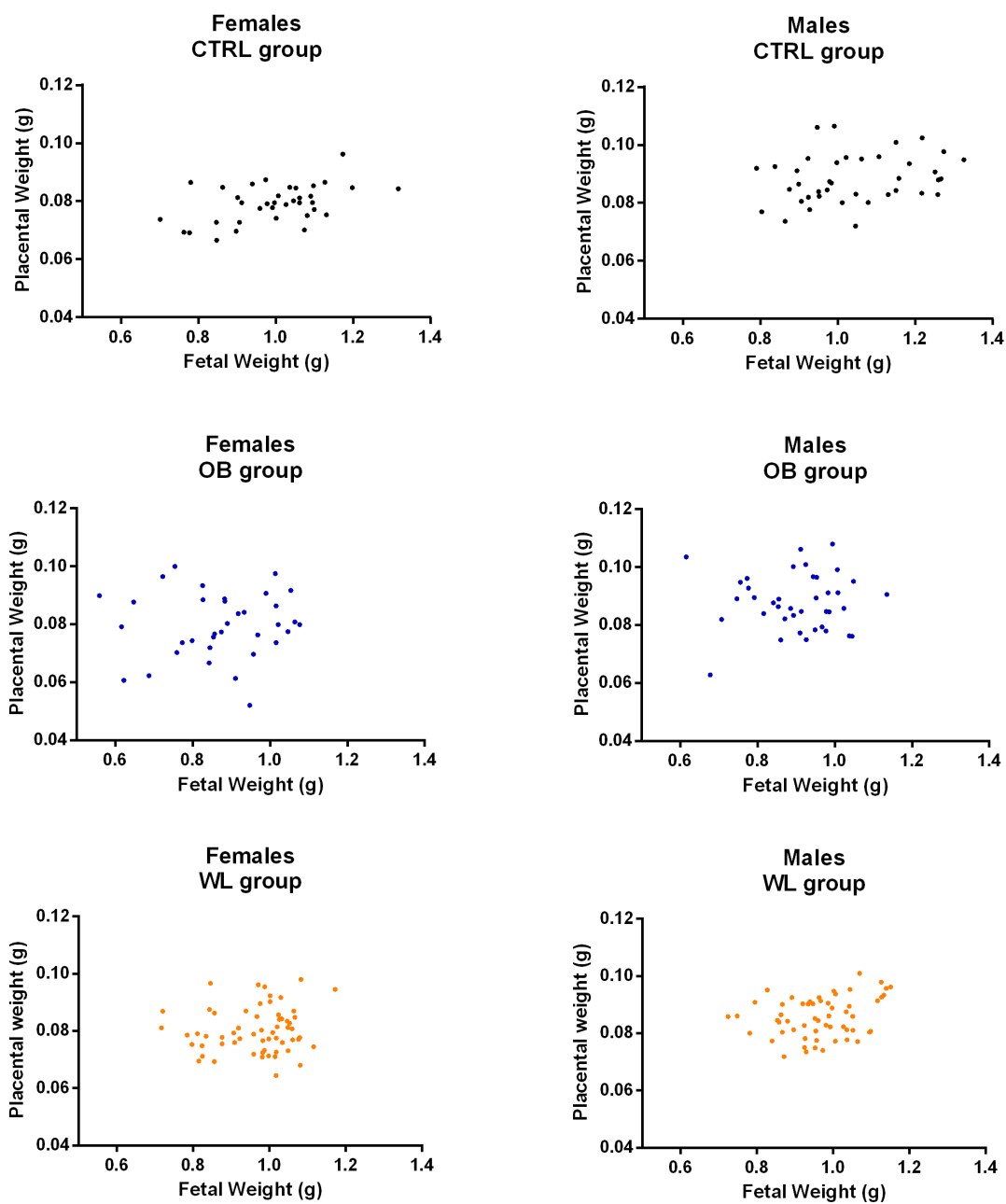

Supplement: Additional file 1: — Supplementary Figures S1 to S6. Food intake (FI) to body weight (BW) ratio in females during the preconceptional period. (a) P < 0.05 OB vs. CTRL, (b) P < 0.05 WL vs. CTRL, (c) P < 0.05 WL vs. OB. n = 18–20 CTRL, 23 OB, 17–19 WL. Figure S2. Fetal weight as a function of litter size in dams at E18.5. Both sexes were combined as there was no effect of sex on fetal weight. Figure S3. Placental weight as a function of litter size and sex at E18.5. Figure S4. Effect of maternal age on fetal parameters. (A) Relationship between maternal age and fetal weight. (B) Relationship between maternal age and fetal-weight-to-placental-weight ratio index (FPI). For statistical analysis, see text. Figure S5. Distribution of fetal weight in CTRL, OB, and WL dams at E18.5. CTRL dams are represented in black, WL dams in brown, and OB dams in blue. The red line represents the 10th percentile of CTRL population. Figure S6. Relationship between fetal and placental weight in female and male offspring at E18.5. For statistical analysis, see text and “Methods” section. M: males, F: females. [file 13148_2016_188_MOESM1_ESM.pdf]
