# Supplementary material for: Expression of epigenetic machinery genes is sensitive to maternal obesity and weight loss in relation to fetal growth in mice
Source: Clin Epigenetics. 2016 Feb 27;8:22. doi: 10.1186/s13148-016-0188-3 (PMC4769534; doi:10.1186/s13148-016-0188-3)
Supplement: Additional file 3: — Description of the selection criteria of genes for the custom TLDA design and related bibliographic references. [file 13148_2016_188_MOESM3_ESM.pdf]

Design of the Custom TaqMan Gene Expression Array Cards

We hypothesized that maternal weight trajectories could alter the expression of epigenetic machinery genes or genes implicated in metabolism or in development. The originality of this study was that different families of epigenetic modifiers were examined. Based on literature and our previous study [1], 60 epigenetic genes were selected because of their implication in metabolic processes, fetal growth, obesity, type-2 diabetes, developmental conditioning of offspring phenotype or the response to maternal nutrition. The second aim of this expression study was to assess the expression of known target genes of developmental conditioning by maternal nutrition (32 genes):

1) DNA methylation

DNA methyltransferases are implicated in developmental programming, fetal growth restriction or the response to maternal HFD [1–6]. We also studied the genes coding for **DNA hydroxymethylation** enzymes (TETs) to see if maternal metabolism could have an impact of their expression. **Methyl-binding domain-containing proteins** play an important role in brain development and in mental disorders, which associate with alterations in feeding behaviour, and also in glucose homeostasis [2,4,7–14].

2) Histone methylation

Some **lysine methyltransferases** are involved in obesity, diabetes, adipogenesis and the response to maternal HFD [1,15–20]. **Lysine demethylases** are also involved in obesity [1,21–23]. **Arginine transferases** are known regulators of glucose metabolism and adipogenesis [24–28].

3) Histone acetylation

Genes related to histone acetylation were particularly suitable to our selection criterion; thus, 29 members of these families were studied out of 60 epigenetic machinery genes. **Histone deacetylases** are involved in adipogenesis, fetal growth restriction and developmental programming by maternal nutrition [5,6,29–32]. **Sirtuins** are largely implicated in lipid and glucose metabolism, and are important sensors of caloric restriction [33–39]. Some sirtuins are associated with an obese phenotype [40–45]. Many members of the **lysine acetyltransferase** family play a role in metabolic diseases like obesity, diabetes and hepatic steatosis [46–55]. **Bromodomain proteins** 2 and 4 are involved in obesity and in adipogenesis [56–58].

4) Glucose and lipid metabolism: *Pparα, Pparγ, Pparδ*, *Pepck*, *Pgc-1α*, *C/Ebp-α, C/Ebp-β*, *Gck*, *Lpl*, *Rev-erbα*, *Nocturnin*, *OxtR*

5) Serotonin signaling: *Tph1*, *Slc6a4*, *MaoA*, *5-HT-r2a*, *5-HT-r2c*

6) Glucocorticoid signaling and metabolism: *11βHsd-1*, *11βHsd-2*, *Gr*

7) Appetite regulation: *leptin*, *leptin receptor*, *Pomc*, *Npy*, *Bdnf*,

8) Feto-placental growth: *Gcm1*, *GcGr*, *Igf2*, *Igf2r*, *Slc16a10*, *Irs-1*, *InsR*

For the first three categories, we chose all members of the epigenetic machinery for which we found information related to our selection criterion. Genes for which we did not find relevant information or that have never been studied in the context of obesity were not taken into

account: *Kdm1a*, *Mbd4*, *Brd4*, *Prmt3*, *Prmt4*, *Prmt6*, *Phf8*, *Kdm4b*, *Kdm4c*, *Kdm5b*, *Jarid2*, *Kmt2a*, *Kmt2b*, *Kmt2c*, *Kmt2d*, *Kmt3b*, *Kmt5a*, *Kmt6*.

For a subset of genes in categories 4 to 8, epigenetic alterations are documented in the context of developmental conditioning. For example, methylation and histone modifications in the promoter of the ***glucocorticoid receptor*** are altered in response to maternal nutrition or behaviour [4,59–63]. Methylation of the ***leptin*** promoter and the anorexygenic hypothalamic neuropeptide ***Pomc*** promoter is modified by maternal high-fat or low-protein diet [64–67]. The maternal nutritional environment alters epigenetic marks at the level of transcriptional factors involved in the development of metabolic tissues [60,68–73].

All genes assessed in this study are listed in Additional file 2 with their assay ID numbers (Table S1).

REFERENCES:

1. Gabory A, Ferry L, Fajardy I, Jouneau L, Gothié J-D, Vigé A, et al. Maternal Diets Trigger Sex-Specific Divergent Trajectories of Gene Expression and Epigenetic Systems in Mouse Placenta. Aguila MB, editor. PLoS ONE. 2012;7:e47986.

2. Ganguly A, Chen Y, Shin B-C, Devaskar SU. Prenatal caloric restriction enhances DNA methylation and MeCP2 recruitment with reduced murine placental glucose transporter isoform 3 expression. J. Nutr. Biochem. 2014;25:259–66.

3. Jensen Peña C, Monk C, Champagne FA. Epigenetic Effects of Prenatal Stress on 11β-Hydroxysteroid Dehydrogenase-2 in the Placenta and Fetal Brain. Sun K, editor. PLoS ONE. 2012;7:e39791.

4. Lillycrop KA, Slater-Jefferies JL, Hanson MA, Godfrey KM, Jackson AA, Burdge GC. Induction of altered epigenetic regulation of the hepatic glucocorticoid receptor in the offspring of rats fed a protein-restricted diet during pregnancy suggests that reduced DNA methyltransferase-1 expression is involved in impaired DNA methylation and changes in histone modifications. Br. J. Nutr. 2007;97:1064.

5. Park JH, Stoffers DA, Nicholls RD, Simmons RA. Development of type 2 diabetes following intrauterine growth retardation in rats is associated with progressive epigenetic silencing of Pdx1. J. Clin. Invest. 2008;118:2316–24.

6. Raychaudhuri N, Raychaudhuri S, Thamotharan M, Devaskar SU. Histone Code Modifications Repress Glucose Transporter 4 Expression in the Intrauterine Growth-restricted Offspring. J. Biol. Chem. 2008;283:13611–26.

7. Adegbola AA, Gonzales ML, Chess A, LaSalle JM, Cox GF. A novel hypomorphic MECP2 point mutation is associated with a neuropsychiatric phenotype. Hum. Genet. 2009;124:615–23.

8. Bonnet C, Khan AA, Bresso E, Vigouroux C, Béri M, Lejczak S, et al. Extended spectrum of MBD5 mutations in neurodevelopmental disorders. Eur. J. Hum. Genet. 2013;21:1457–61.

9. Du Y, Liu B, Guo F, Xu G, Ding Y, Liu Y, et al. The Essential Role of Mbd5 in the Regulation of Somatic Growth and Glucose Homeostasis in Mice. Chowen JA, editor. PLoS ONE. 2012;7:e47358.

10. Fyffe SL, Neul JL, Samaco RC, Chao H-T, Ben-Shachar S, Moretti P, et al. Deletion of Mecp2 in Sim1-expressing neurons reveals a critical role for MeCP2 in feeding behavior, aggression, and the response to stress. Neuron. 2008;59:947–58.

11. Hendrich B. Closely related proteins MBD2 and MBD3 play distinctive but interacting roles in mouse development. Genes Dev. 2001;15:710–23.

12. Samaco RC, Fryer JD, Ren J, Fyffe S, Chao H-T, Sun Y, et al. A partial loss of function allele of Methyl-CpG-binding protein 2 predicts a human neurodevelopmental syndrome. Hum. Mol. Genet. 2008;17:1718–27.

13. Sitras V, Paulssen R, Leirvik J, Vartun A, Acharya G. Placental Gene Expression Profile in Intrauterine Growth Restriction Due to Placental Insufficiency. Reprod. Sci. 2009;16:701–11.

14. Zhao X, Ueba T, Christie BR, Barkho B, McConnell MJ, Nakashima K, et al. Mice lacking methyl-CpG binding protein 1 have deficits in adult neurogenesis and hippocampal function. Proc. Natl. Acad. Sci. 2003;100:6777–82.

15. Kleefstra T, Kramer JM, Neveling K, Willemsen MH, Koemans TS, Vissers LELM, et al. Disruption of an EHMT1-Associated Chromatin-Modification Module Causes Intellectual Disability. Am. J. Hum. Genet. 2012;91:73–82.

16. Lohmann F, Loureiro J, Su H, Fang Q, Lei H, Lewis T, et al. KMT1E Mediated H3K9 Methylation is Required for the Maintenance of Embryonic Stem Cells by Repressing Trophectoderm Differentiation. STEM CELLS [Internet]. 2009 [cited 2015 Nov 19]; Available from: http://doi.wiley.com/10.1002/stem.278

17. Okabe J, Orlowski C, Balcerczyk A, Tikellis C, Thomas MC, Cooper ME, et al. Distinguishing Hyperglycemic Changes by Set7 in Vascular Endothelial Cells. Circ. Res. 2012;110:1067–76.

18. Okamura M, Inagaki T, Tanaka T, Sakai J. Role of histone methylation and demethylation in adipogenesis and obesity. Organogenesis. 2010;6:24–32.

19. Villeneuve LM, Kato M, Reddy MA, Wang M, Lanting L, Natarajan R. Enhanced Levels of microRNA-125b in Vascular Smooth Muscle Cells of Diabetic db/db Mice Lead to Increased Inflammatory Gene Expression by Targeting the Histone Methyltransferase Suv39h1. Diabetes. 2010;59:2904–15.

20. Xin Z, Tachibana M, Guggiari M, Heard E, Shinkai Y, Wagstaff J. Role of Histone Methyltransferase G9a in CpG Methylation of the Prader-Willi Syndrome Imprinting Center. J. Biol. Chem. 2003;278:14996–5000.

21. Gupta J, Kumar S, Li J, Krishna Murthy Karuturi R, Tikoo K. Histone H3 lysine 4 monomethylation (H3K4me1) and H3 lysine 9 monomethylation (H3K9me1): distribution and their association in regulating gene expression under hyperglycaemic/hyperinsulinemic conditions in 3T3 cells. Biochimie. 2012;94:2656–64.

22. Okada Y, Tateishi K, Zhang Y. Histone Demethylase JHDM2A Is Involved in Male Infertility and Obesity. J. Androl. 2010;31:75–8.

23. Tateishi K, Okada Y, Kallin EM, Zhang Y. Role of Jhdm2a in regulating metabolic gene expression and obesity resistance. Nature. 2009;458:757–61.

24. Choi D, Oh K-J, Han H-S, Yoon Y-S, Jung C-Y, Kim S-T, et al. Protein arginine methyltransferase 1 regulates hepatic glucose production in a FoxO1-dependent manner. Hepatology. 2012;56:1546–56.

25. Han H-S, Choi D, Choi S, Koo S-H. Roles of Protein Arginine Methyltransferases in the Control of Glucose Metabolism. Endocrinol. Metab. 2014;29:435.

26. Iwasaki H, Kovacic JC, Olive M, Beers JK, Yoshimoto T, Crook MF, et al. Disruption of Protein Arginine N-Methyltransferase 2 Regulates Leptin Signaling and Produces Leanness In Vivo Through Loss of STAT3 Methylation. Circ. Res. 2010;107:992–1001.

27. LeBlanc SE, Konda S, Wu Q, Hu Y-J, Oslowski CM, Sif S, et al. Protein Arginine Methyltransferase 5 (Prmt5) Promotes Gene Expression of Peroxisome Proliferator-Activated Receptor γ2 (PPARγ2) and Its Target Genes during Adipogenesis. Mol. Endocrinol. 2012;26:583–97.

28. Yamagata K, Daitoku H, Takahashi Y, Namiki K, Hisatake K, Kako K, et al. Arginine methylation of FOXO transcription factors inhibits their phosphorylation by Akt. Mol. Cell. 2008;32:221–31.

29. Aagaard-Tillery KM, Grove K, Bishop J, Ke X, Fu Q, McKnight R, et al. Developmental origins of disease and determinants of chromatin structure: maternal diet modifies the primate fetal epigenome. J. Mol. Endocrinol. 2008;41:91–102.

30. Fu Q. Uteroplacental insufficiency induces site-specific changes in histone H3 covalent modifications and affects DNA-histone H3 positioning in day 0 IUGR rat liver. Physiol. Genomics. 2004;20:108–16.

31. Yoo EJ, Chung J-J, Choe SS, Kim KH, Kim JB. Down-regulation of Histone Deacetylases Stimulates Adipocyte Differentiation. J. Biol. Chem. 2006;281:6608–15.

32. Zheng S, Li Q, Zhang Y, Balluff Z, Pan Y-X. Histone deacetylase 3 (HDAC3) participates in the transcriptional repression of the p16INK4a gene in mammary gland of the female rat offspring exposed to an early-life high-fat diet. Epigenetics. 2012;7:183–90.

33. Haigis MC, Mostoslavsky R, Haigis KM, Fahie K, Christodoulou DC, Murphy AJ, et al. SIRT4 Inhibits Glutamate Dehydrogenase and Opposes the Effects of Calorie Restriction in Pancreatic β Cells. Cell. 2006;126:941–54.

34. Jing E, Gesta S, Kahn CR. SIRT2 Regulates Adipocyte Differentiation through FoxO1 Acetylation/Deacetylation. Cell Metab. 2007;6:105–14.

35. Kanfi Y, Shalman R, Peshti V, Pilosof SN, Gozlan YM, Pearson KJ, et al. Regulation of SIRT6 protein levels by nutrient availability. FEBS Lett. 2008;582:543–8.

36. Kim H-S, Xiao C, Wang R-H, Lahusen T, Xu X, Vassilopoulos A, et al. Hepatic-Specific Disruption of SIRT6 in Mice Results in Fatty Liver Formation Due to Enhanced Glycolysis and Triglyceride Synthesis. Cell Metab. 2010;12:224–36.

37. Purushotham A, Schug TT, Xu Q, Surapureddi S, Guo X, Li X. Hepatocyte-Specific Deletion of SIRT1 Alters Fatty Acid Metabolism and Results in Hepatic Steatosis and Inflammation. Cell Metab. 2009;9:327–38.

38. Rodgers JT, Puigserver P. Fasting-dependent glucose and lipid metabolic response through hepatic sirtuin 1. Proc. Natl. Acad. Sci. U. S. A. 2007;104:12861–6.

39. Suter MA, Chen A, Burdine MS, Choudhury M, Harris RA, Lane RH, et al. A maternal high-fat diet modulates fetal SIRT1 histone and protein deacetylase activity in nonhuman primates. FASEB J. 2012;26:5106–14.

40. Crujeiras AB, Parra D, Goyenechea E, Martínez JA. Sirtuin gene expression in human mononuclear cells is modulated by caloric restriction. Eur. J. Clin. Invest. 2008;38:672–8.

41. Hirschey MD, Shimazu T, Jing E, Grueter CA, Collins AM, Aouizerat B, et al. SIRT3 Deficiency and Mitochondrial Protein Hyperacetylation Accelerate the Development of the Metabolic Syndrome. Mol. Cell. 2011;44:177–90.

42. Nasrin N, Wu X, Fortier E, Feng Y, Bare' OC, Chen S, et al. SIRT4 Regulates Fatty Acid Oxidation and Mitochondrial Gene Expression in Liver and Muscle Cells. J. Biol. Chem. 2010;285:31995–2002.

43. Savastano S, Di Somma C, Colao A, Barrea L, Orio F, Finelli C, et al. Preliminary data on the relationship between circulating levels of Sirtuin 4, anthropometric and metabolic parameters in obese subjects according to growth hormone/insulin-like growth factor-1 status. Growth Horm. IGF Res. 2015;25:28–33.

44. Schwer B, Schumacher B, Lombard DB, Xiao C, Kurtev MV, Gao J, et al. Neural sirtuin 6 (Sirt6) ablation attenuates somatic growth and causes obesity. Proc. Natl. Acad. Sci. 2010;107:21790–4.

45. Yoshizawa T, Karim MF, Sato Y, Senokuchi T, Miyata K, Fukuda T, et al. SIRT7 Controls Hepatic Lipid Metabolism by Regulating the Ubiquitin-Proteasome Pathway. Cell Metab. 2014;19:712–21.

46. Bricambert J, Miranda J, Benhamed F, Girard J, Postic C, Dentin R. Salt-inducible kinase 2 links transcriptional coactivator p300 phosphorylation to the prevention of ChREBP-dependent hepatic steatosis in mice. J. Clin. Invest. 2010;120:4316–31.

47. Coste A, Louet J-F, Lagouge M, Lerin C, Antal MC, Meziane H, et al. The genetic ablation of SRC-3 protects against obesity and improves insulin sensitivity by reducing the acetylation of PGC-1α. Proc. Natl. Acad. Sci. 2008;105:17187–92.

48. Garaulet M, Corbalan MD, Madrid JA, Morales E, Baraza JC, Lee Y-C, et al. CLOCK gene is implicated in weight reduction in obese patients participating in a dietary programme based on the Mediterranean diet. Int. J. Obes. 2010;34:516–23.

49. Lerin C, Rodgers JT, Kalume DE, Kim S, Pandey A, Puigserver P. GCN5 acetyltransferase complex controls glucose metabolism through transcriptional repression of PGC-1α. Cell Metab. 2006;3:429–38.

50. Oishi K, Shirai H, Ishida N. CLOCK is involved in the circadian transactivation of peroxisome-proliferator-activated receptor alpha (PPARalpha) in mice. Biochem J. 2005;386:575–81.

51. Picard F, Géhin M, Annicotte J-S, Rocchi S, Champy M-F, O'Malley BW, et al. SRC-1 and TIF2 control energy balance between white and brown adipose tissues. Cell. 2002;111:931–41.

52. Sookoian S, Gemma C, Gianotti TF, Burgueño A, Castaño G, Pirola CJ. Genetic variants of Clock transcription factor are associated with individual susceptibility to obesity. Am. J. Clin. Nutr. 2008;87:1606–15.

53. Turek FW. Obesity and Metabolic Syndrome in Circadian Clock Mutant Mice. Science. 2005;308:1043–5.

54. Wang Z, Qi C, Krones A, Woodring P, Zhu X, Reddy JK, et al. Critical roles of the p160 transcriptional coactivators p/CIP and SRC-1 in energy balance. Cell Metab. 2006;3:111–22.

55. Zhou XY, Shibusawa N, Naik K, Porras D, Temple K, Ou H, et al. Insulin regulation of hepatic gluconeogenesis through phosphorylation of CREB-binding protein. Nat. Med. 2004;10:633–7.

56. Couture J-P, Nolet G, Beaulieu E, Blouin R, Gévry N. The p400/Brd8 chromatin remodeling complex promotes adipogenesis by incorporating histone variant H2A.Z at PPARγ target genes. Endocrinology. 2012;153:5796–808.

57. Denis GV. Bromodomain coactivators in cancer, obesity, type 2 diabetes, and inflammation. Discov. Med. 2010;10:489.

58. Wang F, Liu H, Blanton WP, Belkina A, Lebrasseur NK, Denis GV. *Brd2* disruption in mice causes severe obesity without Type 2 diabetes. Biochem. J. 2010;425:71–83.

59. Begum G, Davies A, Stevens A, Oliver M, Jaquiery A, Challis J, et al. Maternal Undernutrition Programs Tissue-Specific Epigenetic Changes in the Glucocorticoid Receptor in Adult Offspring. Endocrinology. 2013;154:4560–9.

60. Burdge GC, Slater-Jefferies J, Torrens C, Phillips ES, Hanson MA, Lillycrop KA. Dietary protein restriction of pregnant rats in the F0 generation induces altered methylation of hepatic gene promoters in the adult male offspring in the F1 and F2 generations. Br. J. Nutr. 2007;97:435.

61. Conradt E, Lester BM, Appleton AA, Armstrong DA, Marsit CJ. The roles of DNA methylation of *NR3C1* and *11β-HSD2* and exposure to maternal mood disorder in utero on newborn neurobehavior. Epigenetics. 2013;8:1321–9.

62. Oberlander TF, Weinberg J, Papsdorf M, Grunau R, Misri S, Devlin AM. Prenatal exposure to maternal depression, neonatal methylation of human glucocorticoid receptor gene (NR3C1) and infant cortisol stress responses. Epigenetics. 2008;3:97–106.

63. Weaver ICG, Cervoni N, Champagne FA, D'Alessio AC, Sharma S, Seckl JR, et al. Epigenetic programming by maternal behavior. Nat. Neurosci. 2004;7:847–54.

64. Jousse C, Parry L, Lambert-Langlais S, Maurin A-C, Averous J, Bruhat A, et al. Perinatal undernutrition affects the methylation and expression of the leptin gene in adults: implication for the understanding of metabolic syndrome. FASEB J. 2011;25:3271–8.

65. Plagemann A, Harder T, Brunn M, Harder A, Roepke K, Wittrock-Staar M, et al. Hypothalamic proopiomelanocortin promoter methylation becomes altered by early overfeeding: an epigenetic model of obesity and the metabolic syndrome: Nutritionally induced alterations of POMC promoter methylation. J. Physiol. 2009;587:4963–76.

66. Tobi EW, Lumey LH, Talens RP, Kremer D, Putter H, Stein AD, et al. DNA methylation differences after exposure to prenatal famine are common and timing- and sex-specific. Hum. Mol. Genet. 2009;18:4046–53.

67. Zheng J, Xiao X, Zhang Q, Yu M, Xu J, Wang Z, et al. Maternal and post-weaning high-fat, high-sucrose diet modulates glucose homeostasis and hypothalamic POMC promoter methylation in mouse offspring. Metab. Brain Dis. [Internet]. 2015 [cited 2015 Jun 10]; Available from: http://link.springer.com/10.1007/s11011-015-9678-9

68. Borengasser SJ, Zhong Y, Kang P, Lindsey F, Ronis MJJ, Badger TM, et al. Maternal Obesity Enhances White Adipose Tissue Differentiation and Alters Genome-Scale DNA Methylation in Male Rat Offspring. Endocrinology. 2013;154:4113–25.

69. Borengasser SJ, Kang P, Faske J, Gomez-Acevedo H, Blackburn ML, Badger TM, et al. High Fat Diet and In Utero Exposure to Maternal Obesity Disrupts Circadian Rhythm and Leads to Metabolic Programming of Liver in Rat Offspring. Sookoian SC, editor. PLoS ONE. 2014;9:e84209.

70. Lillycrop KA, Phillips ES, Torrens C, Hanson MA, Jackson AA, Burdge GC. Feeding pregnant rats a protein-restricted diet persistently alters the methylation of specific cytosines in the hepatic PPARα promoter of the offspring. Br. J. Nutr. [Internet]. 2008 [cited 2015 Jun 22];100. Available from: http://www.journals.cambridge.org/abstract_S0007114507894438

71. Pruis MGM, Lendvai á., Bloks VW, Zwier MV, Baller JFW, de Bruin A, et al. Maternal western diet primes non-alcoholic fatty liver disease in adult mouse offspring. Acta Physiol. 2014;210:215–27.

72. Strakovsky RS, Zhang X, Zhou D, Pan Y-X. Gestational high fat diet programs hepatic phosphoenolpyruvate carboxykinase gene expression and histone modification in neonatal offspring rats: Programming gluconeogenesis by gestational high fat diet. J. Physiol. 2011;589:2707–17.

73. Zheng S, Rollet M, Pan Y-X. Maternal protein restriction during pregnancy induces CCAAT/enhancer-binding protein (C/EBPβ) expression through the regulation of histone modification at its promoter region in female offspring rat skeletal muscle. Epigenetics. 2011;6:161–70.
